# Evaluation of Broad-Spectrum Pesticides Based on Unified Multi-Analytical Procedure in Fruits and Vegetables for Acute Health Risk Assessment

**DOI:** 10.3390/foods14142528

**Published:** 2025-07-18

**Authors:** Bożena Łozowicka, Piotr Kaczyński, Magdalena Jankowska, Ewa Rutkowska, Piotr Iwaniuk, Rafał Konecki, Weronika Rogowska, Aida Zhagyparova, Damira Absatarova, Stanisław Łuniewski, Marcin Pietkun, Izabela Hrynko

**Affiliations:** 1 Institute of Plant Protection—National Research Institute, Chełmońskiego 22 St., 15-195 Białystok, Polandw.rogowska@iorpib.poznan.pl (W.R.);; 2 Faculty of Economics, L.N. Gumilyov Eurasian National University, Satbayev 2 St., 010008 Astana, Kazakhstan; 3 Faculty of Natural and Technical Sciences, Zhetysu University, Zhansugurov 187A St., 040009 Taldykorgan, Kazakhstan; 4 Eastern European University of Applied Sciences in Białystok, Ciepła 40 St., 15-472 Białystok, Poland; 5 Hydratec, Radziwonika 12 St., 15-166 Białystok, Poland

**Keywords:** pesticide residues, fruit, vegetable, MRL, health risk assessment, unapproved pesticides

## Abstract

Fruits and vegetables are crucial components of a healthy diet, which are susceptible to pests. Therefore, the application of pesticides is a basic manner of crop chemical protection. The aim of this study was a comprehensive analysis of pesticide occurrence in 1114 samples of fruits and vegetables. A unified multi-analytical protocol was used composed of primary–secondary amine/graphitized carbon black/magnesium sulfate to purify samples with diversified profile of interfering substances. Moreover, the obtained analytical data were used to evaluate the critical acute health risk in subpopulations of children and adults within European limits criteria. Out of 550 pesticides analyzed, 38 and 69 compounds were noted in 58.6% of fruits and 44.2% of vegetables, respectively. Acetamiprid (14.1% of all detections) and captan (11.3%) occurred the most frequently in fruits, while pendimethalin (10.6%) and azoxystrobin (8.6%) occurred the most frequently in vegetables. A total of 28% of vegetable and 43% of fruit samples were multiresidues with up to 13 pesticides in dill, reaching a final concentration of 0.562 mg kg^−1^. Maximum residue level (MRL) was exceeded in 7.9% of fruits and 7.3% of vegetables, up to 7900% MRL for chlorpyrifos in dill (0.79 mg kg^−1^). Notably, 8 out of 38 pesticides found in fruits (21%; 1.2% for carbendazim) and 24 out of 69 compounds in vegetables (35%, 7.4% for chlorpyrifos) were not approved in the EU. Concentrations of pesticides exceeding MRL were used to assess acute health risk for children and adults. Moreover, the incidence of acute health risk was proved for children consuming parsnip with linuron (156%). In other cases, it was below 100%, indicating that Polish food is safe. The work provides reliable and representative scientific data on the contamination of fruits and vegetables with pesticides. It highlights the importance of legislative changes to avoid the occurrence of not approved pesticides in the EU, increasing food and health safety.

## 1. Introduction

The global production and consumption of fruit and vegetables is constantly on the rise due to the increasingly common adoption of healthy nutritional habits. The presence of fruit and vegetables in the diet provides the organism with a range of key bioactive substances (including flavonoids, anthocyanins, tannins, etc.) that provide health benefits [1,2]; among other things, they show anti-cancer and antioxidation properties and prevent diabetes and circulatory diseases [3,4]. According to the recommendations by the World Health Organization, it is worth taking five servings of vegetables and fruit every day [5].

However, in addition to their health properties, fruits and vegetables can be a potential source of harmful substances, such as pesticides. These products are one of the routes of exposure to pesticide residues because their cultivation requires the use of intensive chemical protection to combat fungal pathogens, pests, and weeds [6,7].

The occurrence of pesticides in fruits and vegetables is a big problem globally, confirmed by many scientific studies. Montiel-Leon et al. [8] indicated that 47% of fruits and vegetables from Canada and USA contained pesticide residues. Pesticides were detected in 65% of fruits and vegetables from Argentina [9]. Moreover, 62% of fruits and vegetables from Turkey contained pesticides [10] and 51% of samples from Poland were contaminated by these compounds [11]. Therefore, scientific studies are pivotal to support monitoring official control of pesticide residues in fruits and vegetables to ensure consumer’s health.

Due to the high frequency of use and various properties, pesticides can, even at low concentrations, have a negative impact on the human organism and contribute to various diseases [12]. Some pesticides have carcinogenic, genotoxic, neurotoxic, teratogenic, and endocrine- and reproductive system-disrupting effects [13]. Daily exposure even to small doses contributes to the development of chronic diseases, especially neurodegenerative diseases such as Alzheimer’s disease, Parkinson’s disease, and amyotrophic lateral sclerosis [14], as well as affects developmental disorders (attention deficit hyperactivity disorder, autism) [15] and metabolic disorders (diabetes, obesity) [16]. Most of these disorders are caused by insecticides and herbicides, in particular organophosphates, organochlorines, phenoxyacetic acids, and triazine compounds [15]. Even though many of the compounds responsible for these effects have been withdrawn in the European Union (EU), including chlorpyrifos, DDT, carbendazim, thiamethoxam, and dicloran, they are still detected in agricultural crops [17]. The concentrations of detected pesticides in food products are particularly important for the health risk assessment of consumers. It is therefore necessary to ensure proper protection of human health by conducting scientific research on the occurrence of pesticides in agricultural crops and the correctness of their use, supporting official control.

Accurate, sensitive, and solid analytical procedures are of key importance in the analysis of pesticide levels in food. The available literature shows a trend towards the development of quick methods of simultaneous identification of residues of multiple ingredients, often representing different chemical classes [17,18,19,20,21]. This necessity results, among other things, from the use of diverse active substances, which may cause co-occurrence in foods of a wide range of pesticides with differing chemical properties and mechanisms of action [17,22,23,24]. They are often connected with the presence of complex matrices in samples, containing compounds that may interfere with the analytes, thus affecting the quality of the final result [25]. In the group of fruit and vegetables, one can distinguish, among other things, products with high water content (e.g., cucumbers, tomatoes, potatoes); products with high acidity and high water content (e.g., strawberries, raspberries, grapes); or products with a high fat content (e.g., avocados or olives). These assortments are rich in interfering substances, i.e., sugars, proteins, lipids, carotenoids, or chlorophyll [26,27,28]. In recent years, the QuEChERS (Quick, Easy, Cheap, Effective, Rugged, and Safe) method, combining solvent extraction with extract purification using the d-SPE technique, has become the most commonly used analytical technique in laboratories worldwide [22,29,30,31]. Under this method, for fatty matrices such as avocados or olives, it is recommended to add C18 (octadecyl) to the extract to remove fatty acids [32,33]. PSA (primary–secondary amine) is used for the removal of sugars, organic acids, fatty acids, and certain pigments [34]. GCB (graphitized carbon black) finds application in the removal of polyphenols, pigments, and dyes [27,35,36,37,38]. However, its strong affinity to flat molecules makes it unsuitable for the purification of extracts during the identification of compounds adsorbing to GCB [39].

The objective of the study was (i) the development of a precise clean-up analytical method using the GC/LC/MS/MS technique, enabling identification of a wide range of pesticides in fruit and vegetable matrices with diverse content of interfering substances: sugars, lipids, proteins, dyes; (ii) comprehensive assessment of pesticide residue levels in fruit and vegetable samples in the 2-year cycle (2023–2024) in relation to the maximum residue limits (MRLs) and incoherent application with European legislation; (iii) critical estimation of short-term exposure as well as characterization of the related consumer acute health risk for diversified subpopulations of children and adults.

## 2. Materials and Methods

### 2.1. Pesticides

Pesticide reference standards with certified purity of >99% were obtained from Dr. Ehrenstorfer Laboratory (Augsburg, Germany). The analytical studies covered 550 pesticides and their metabolites, as well as degradation products, belonging to five biological groups: acaricides (11), fungicides (142), herbicides (157), insecticides (233), growth regulators (4), and others (3). The intention of selection these pesticides was to include a wide range of available compounds, approved and not approved in the EU, to achieve the most reliable picture of pesticide occurrence in fruits and vegetables.

Triphenyl phosphate (TPP), atrazine-d5, carbendazim-d3, and isoproturon-d6 as the internal standards (IS) were supplied from Sigma-Aldrich (St. Louis, MO, USA).

### 2.2. Reagents and Materials

Formic acid (98–100%), isopropanol (LC/MS), acetonitrile, and methanol (high-performance liquid chromatography-grade) were purchased from Merck (Darmstadt, Germany). Acetone, acetonitrile, and n-hexane were sourced from J.T. Baker (Deventer, The Netherlands). Deionized water was obtained using an automatic purification system (Millipore Ltd., Bedford, MA, USA). QuEChERS kits and dispersive solid-phase extraction (d-SPE) sorbents containing primary–secondary amine (PSA), graphitized carbon black (GCB), and magnesium sulfate (MgSO_4_) were sourced from Agilent Technologies (Santa Clara, CA, USA).

### 2.3. Samples

A total of 1114 samples, of which 370 fruit and 744 vegetable samples most frequently consumed in Poland, were the subject of this research and were taken under Polish official control in 2023–2024 from 16 voivodships throughout Poland. Examined samples were classified in 10 groups by utility features. The assortment groups identified in the 2-year cycle were (1) berries and small fruits (n = 179), (2) stone fruits (n= 168), (3) pome fruits (n = 23), (4) bulb vegetables (n = 17), (5) brassicas (n= 36), (6) root and tuber vegetables (n = 166), (7) leaf vegetables and herbs (n = 182), (8) stem vegetables (n= 85), (9) fruiting vegetables (n = 174), and (10) leguminous vegetables (n = 84) (Table 1).

About 2 kg of fruit/vegetable samples (0.5 kg in the case of leafy vegetables and herbs) were collected from open fields and immediately transported to the laboratory. Then, samples were homogenized, divided into analytical subsamples, and stored in a refrigerator before analysis.

### 2.4. Sample Preparation

**Figure 1:**
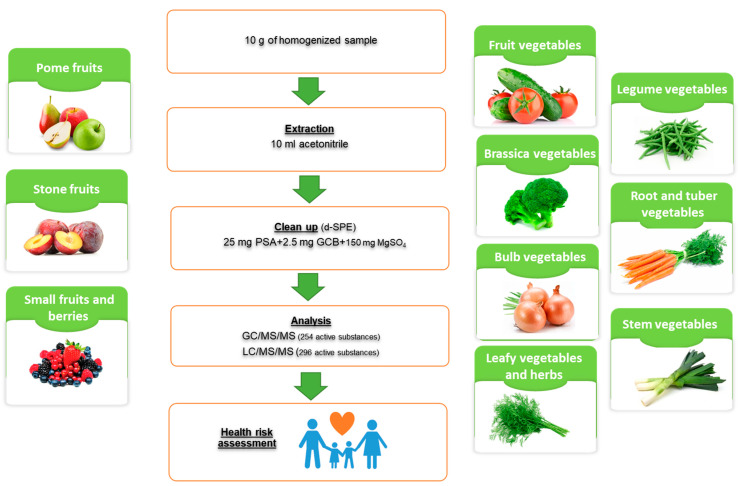
Scheme of fruit and vegetable sample preparation using chromatographic methods.

The fruit and vegetable samples were analyzed by the accredited, multiresidue, modified QuEChERS method (Figure 1). The details of sample preparation were described in a previous publication [36]. Briefly, 10 g of sample was mixed with 10 mL of acetonitrile and vortexed. Next, buffering salts were added consisting of 4 g MgSO_4_, 1 g NaCl, 1 g trisodium citrate dihydrate (Na_3_C_6_H_5_O_7_·2H_2_O), and 0.5 g disodium hydrogen citrate sesquihydrate (Na_2_HC_6_H_5_O_7_·1.5H_2_O). The tubes were vortexed and centrifuged. The acetonitrile layer formed at the top of the centrifuge tube was collected and mixed with clean-up sorbent 150 mg MgSO_4_ + 25 mg PSA + 2.5 mg GCB [37]. A 1 mL sample was filtered through a 0.45 PTFE filter, transferred to a chromatographic vial, and subjected to GC/MS/MS and LC/MS/MS.

Pesticide residues were tested using our own multiresidue analytical methods, meeting the requirements of the SANTE guide [38]. Qualitative and quantitative determinations were performed using chromatographic techniques coupled with tandem mass spectrometry (GC/MS/MS, LC/MS/MS) [39,40].

**Table 1:** Assortment groups of fruits and vegetables in relation to interfering substances purified with PSA + GCB + MgSO_4_.

Assortment Groups	Typical Assortment Categories	Typical Representative Assortments	Interfering Substances
FRUITS			
High water content	Pome fruit	Apples, pears	Anthocyanin, proteins, sugars
	Stone fruits	Cherries, plums, sweet cherries	Anthocyanin, proteins, sugars
High acid content and high water content	Small fruits and berries	Blueberries, chokeberries, grapes, raspberries, strawberries	Anthocyanin, sugars
VEGETABLES			
High water content	Brassica vegetables	Broccoli, cauliflowers, head cabbages, kales	Chlorophylls, sulfur compounds
	Bulb vegetables	Garlic, onions	Chlorophylls, sulfur compounds
	Fruit vegetables	Cabbages, cucumbers, pumpkins, tomatoes, zucchini	Carotenoids, lutein, lycopene
	Leafy vegetables and herbs	Dill, lettuces	Carotenoids, chlorophylls, protein
	Legumes vegetables	Broad beans, green beans, green peans, lupine	Carotenoids, chlorophylls, protein
	Root and tuber vegetables	Beetroots, carrots, celeries, horseradishes, parsley roots, parsnips, potatoes	Betalains, carotenoids, chlorophylls, sulfur compounds
	Stem vegetables	Leeks	Chlorophylls, sulfur compounds

PSA + GCB + MgSO_4_: primary–secondary amine + graphitized carbon black + magnesium sulfate.

### 2.5. Instrumentation and Conditions for the GC/LC/MS/MS Techniques

An Agilent 7890A GC and 7000 Series GC/MS/MS system (Agilent, Palo Alto, CA, USA) was used for the separation of 254 GC-amenable analytes and an Eksigent Ultra LC–100 system (Eksigent Technologies, Dublin, CA, USA) supplied with a QTRAP 6500 MS/MS triple quadrupole system (AB Sciex instruments, Foster City, CA, USA) was used for the separation of 296 LC-amenable analytes in fresh fruit and vegetables. In the case of GC–MS/MS, retention time locking (RTL) was used to eliminate the need for adjusting the time segment windows of multiple reaction monitoring (MRM) groups, using chlorpyrifos-methyl as the locking compound at a retention time of 16.593 min. The chromatographic conditions and the triple quadrupole system parameters of the GC- and LC-MS/MS instruments are summarized in Table S1.

Both LC and GC systems were operated in multiple reaction monitoring mode (MRM). Selected reaction monitoring (SRM) experiments were carried out to obtain the maximum sensitivity for the detection of the target molecules. For confirmation of the studied compounds, two SRM transitions and a correct ratio between the abundances of the two optimized SRM transitions (SRM2/SRM1) were used, along with retention time matching. The acquisition parameters are presented in Table S2.

### 2.6. Health Risk Assessment for Children and Adults Consuming Fruits and Vegetables

In this study, the International Estimated Short-Term Intake (IESTI) was calculated for the most critical subpopulations of European children and adults according to the methodology implemented by EFSA Pesticide Residue Intake Model (PRIMo) revision 3.1. [41] for each combination of pesticide residue exceeding MRL and particular fruit/vegetable.

In the acute risk assessment, the algorithm is based on a “worst case” scenario taking into account as input values the high level of consumption at the level of 97.5 percentile (Large Portion, LP) taken from EU Member States for different population age groups combined with the highest residue (HR), which in this study was residue level > MRL in single sample (IESTI) or the current maximum residue level set by European Commission (IESTI_new_).

For acute risk assessment, the values of the acute intake based on the unit weight of raw fruit/vegetable were used followed by various equations. Case 1 for crops with unit weight ≤25 g (e.g., beans, dill, raspberries), case 2a > 25 g where unit weight is below the large portion (apple, plum, tomato), case 2b > 25 g where unit weight is above the large portion (kale, parsnip), and case 3 for food products that are usually bulked or blended before they are consumed (lentil). More details are presented in Table 2.

The short-term dietary exposure was compared to the pesticide acute reference dose (ARfD, mg kg^−1^ bw). For pesticides with an unapplicable ARfD, alternatively, ADI value was used. The short-term intake was expressed as %ARfD calculated from the equation %ARfD = (IESTI or IESTI_new_/ARfD) × 100%. Consumer exposure not exceeding 100% ARfD means health risk assessment was acceptable.

## 3. Results and Discussion

### 3.1. Method Validation Data and Quality Assurance

In the present study, we used the modified and validated multiresidue QuEChERS method to assess the concentration of residues of 550 pesticides in ten different groups of vegetables and fruits. Two complementary instrumental techniques, i.e., LC/MS/MS and GC/MS/MS, were used for pesticide identification [17,20,22].

Clean-up was a crucial step of sample preparation because it enabled removing of interfering substances that negatively affected the instrumental analysis. In our previous scientific investigations, combinations of PSA + C18 + MgSO_4_ and PSA + MgSO_4_ were tested for dry, high-carbohydrate matrices (e.g., cereals) and high-water and acid matrices (e.g., apples) [42,43]. In this study, a combination of GCB with PSA and MgSO_4_ was selected due to the most significant purifying effect for matrices with diversified profile of interfering substances. The three component clean-up ingredients simplified purification step and can be used as universal clean-up mixture for samples characterized by high water and acid content (raspberries); carotenoids content (carrots); sugars and anthocyanin content (sweet cherries); chlorophyll content (dill), lycopene content (tomatoes); sulfur compounds content (leeks); protein and starch content (green beans).

The established GC/LC/MS/MS method was validated for all analyzed compounds and seven matrices that are representative in terms of the content of interfering substances for the respective groups of fruits and vegetables. Therefore, carrot is a representative for the root and tuber vegetables group; dill for the leafy vegetables and herbs group; green beans for the legume vegetables group; leeks for the brassica, bulb, and stem vegetables group; raspberries for the small fruits and berries group; sweet cherries for the stone and pome fruits group; and tomatoes for the fruit vegetables group. The GC/LC/MS/MS method was validated in terms of trueness, precision, selectivity, limits of quantification (LOQ), matrix effect (ME), and measurement uncertainty (U), which fulfill the requirements set by SANTE/11312/2021 guidelines [44]. The samples employed in validation studies did not contain any of the pesticides analyzed.

The performance of the method was assessed by spiking a mixture of 254 pesticides (GC) and 296 (LC) into extracts of fruit and vegetables at three concentrations between 0.005 and 1.0 mg kg^−1^. Almost all the recovery results are within the range of 70–120% except 59 pesticides in vegetables (excluding tomatoes) and 51 in fruit and tomatoes. A similar group of problematic compounds was observed in all matrices analyzed, with recovery values in the range of 60–70% and 120–130%. The fact that the same compounds posed problems in each of the different matrices suggests that extraction conditions, rather than matrix components, were responsible for the low recovery values of these compounds. Of these compounds, acephate and methamidophos are volatile and have the shortest retention times, while captan, dichlofluanid, and tolyfluanid are easily degraded during sample preparation and/or GC injection. Only 1% of the pesticides tested showed recovery values above 120% but below 130%, which could be due to not enough purification of the extract from interfering substances. Relative standard deviations are less than 20%.

LOQ was defined as the lowest validated spike level meeting the method performance acceptability criteria. The LOQ for 87.5% of the pesticides was 0.005 mg kg^−1^ and for 12.5% was 0.01 mg kg^−1^. These values are much smaller or equal than MRLs established by the European Union for fruit and vegetables.

Linearity of the MS/MS systems was evaluated by assessing the signal responses of the target analytes from matrix-matched calibration solutions prepared by spiking blank extracts at five concentration levels, from 0.005 to 1.0 mg kg^−1^. The coefficient of determination (R^2^) was higher than 0.99 for all of the analytes.

Matrix effects (MEs) have the potential to severely interfere with analyte signals owing to the different interfering substances present in the matrix and the properties of pesticides (polarity, thermolability, thermal stability, volatility, molecular weight, or chemical structure). In this pesticide residue analysis, matrix effects were evaluated using the detector response of the standard in the matrix to the detector response of the standard in the solvent. A value >−20% or <20% indicates the absence of matrix effects, a value of <20% indicates signal suppression, and a value of >−20% indicates signal enhancement.

The most of pesticides achieved the ME value in the range of (−20%)–20% for 469 pesticides (85.3% of analyzed pesticides) in carrots, dill, leeks, and green beans and for 530 (96.4%) pesticides in fruit and tomatoes. These results indicated the absence of major matrix effects for pesticides. However, the values of ME for 14.7% of pesticides in vegetables excluding tomatoes and 3.6% for the rest of the analyzed matrices (for example, acephate and acetamiprid, polar compounds; spirodiclofen, a compound with a molecular weight of more than 400 g mol^−1^; and captan, a compound that decomposes at high temperatures) indicated signal suppression or enhancement. Therefore, it is essential to use matrix-matched calibration samples for these pesticides exhibiting obvious matrix effects to permit reliable quantification during pesticide analysis.

The selectivity of the method was ensured by using the MRM mode. Selectivity was confirmed by monitoring transient ions, including one quantifier ion and two qualifier ions, at retention times corresponding to the retention times of the target pesticide residues. The peak area ratio for the qualifier ions to the quantifier ions was within a relative deviation of ±30% from the mean value of the calibration standards in the same sequence.

The uncertainty was determined using a “top-down” empirical model with a confidence coefficient of 95% and taking into account coverage factor k = 2 and was lower than 50% for all pesticides.

All validated parameters are summarized in Table S3. These results showed that the analytical method applied in this work was appropriate for the analysis of targeted pesticide residues in fruit and vegetable samples. Participation in proficiency testing (PT) programs organized by EU Reference Laboratories for pesticide residues was used to confirm the accuracy of the proposed procedure for the quantification of various pesticides in fruits and vegetables. The results of the quality assurance activities were within the acceptable range, and the z-scores (|z-score| < 2) in the PT programs were satisfactory, indicating that the laboratory is technically competent (Table S2).

### 3.2. Pesticides in Fruits and Vegetables

**Figure 2:**
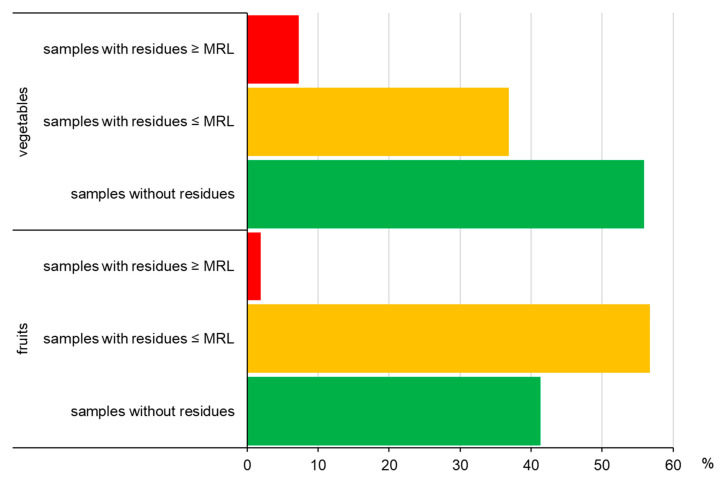
Occurrence of pesticide residues in fruit and vegetable samples.

**Figure 3:**
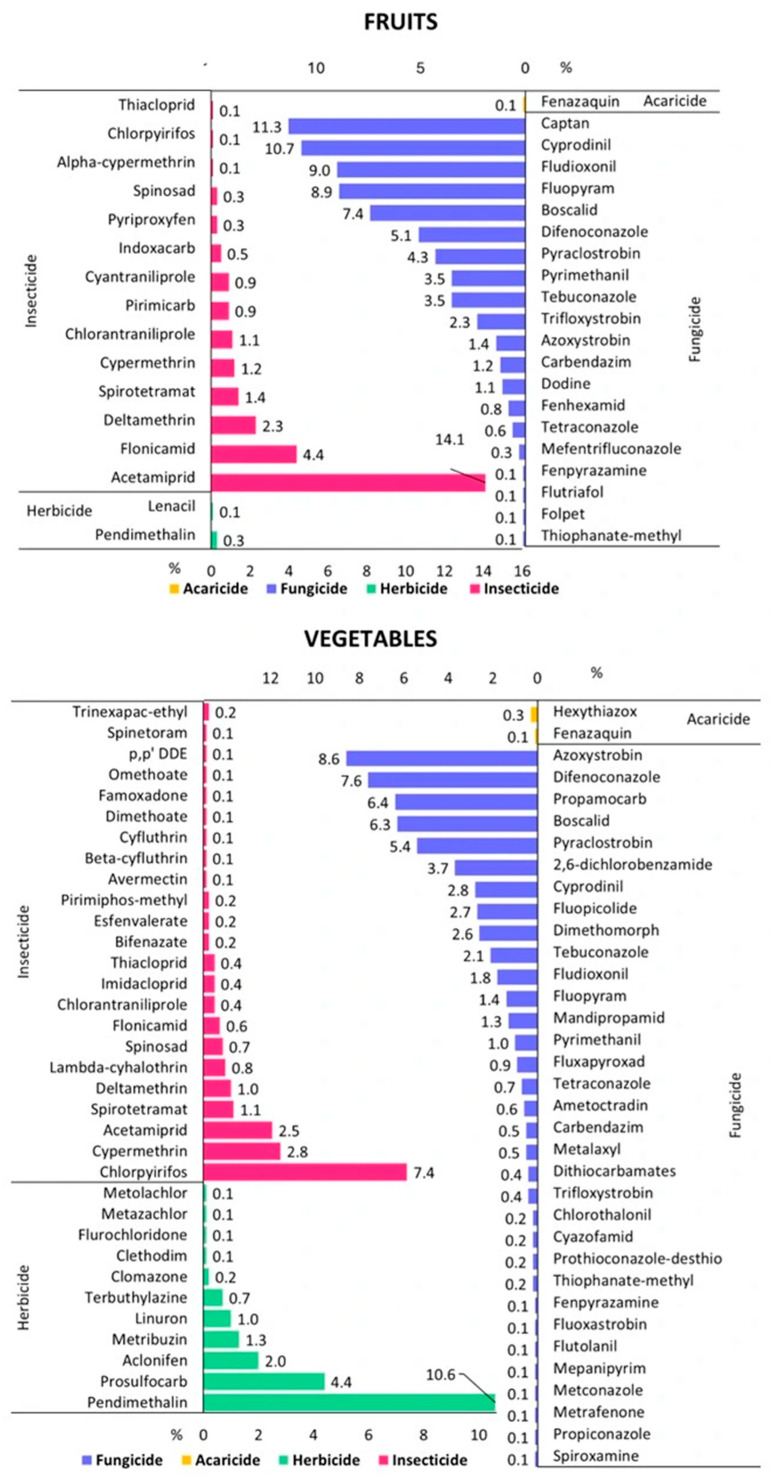
Frequency of detected pesticide residues in fruit and vegetable samples.

The occurrence of pesticide residues in fruits and vegetables is shown in Figure 2. Of the 370 fruit samples tested, 217 contained pesticide residues (58.6%). MRLs were exceeded in 7 samples (1.9%). Indoxacarb in apples exceeded MRL by 160%, flutiafol in raspberries by 200%, and dodine in plums by 420% (Table 3). Out of 550 pesticides analyzed, 38 were detected. The most frequent group of pesticides in fruits were fungicides (20 compounds), followed by insecticides (14), herbicides (2), and acaricide (1). In the group of fungicides, commonly detected were captan (11.3%), cyprodinil (10.7%), fludioxonil (9%), and fluopyram (8.9%). The concentration of fungicides in fruits was in the range of 0.005–6.5 mg kg^−1^, reaching the highest level for captan in cherry (Figure 3). Among insecticides the most frequently determined were acetamiprid (14.1%), flonicamid (4.4%), deltamethrin (2.3%), and spirotetramat (1.4%). Their level varied from 0.005 mg kg^−1^ to 0.45 mg kg^−1^, reaching the greatest content in the case of acetamiprid in cherry. Only two herbicides (pendimethalin, 0.3%, and lenacil, 0.1%) and one acaricide (fenazaquin, 0.1%) were detected in raspberries and apples.

In a previous 3-year study from Poland (2010–2012), pesticides were detected in 50% of fruit samples, while MRL exceedances were noted in 2% of samples [45]. In turn, in fruit tested from the Kuwaiti market, pesticides were detected in 53% of samples in the range from 0.01 to 1.32 mg kg^−1^ [46]. Of these, 37% were below MRL, but 21% exceeded MRL limits. Alokail et al. [47] reported that in fruit sold in Saudi Arabia, only 10 out of 161 samples (6.2%) were pesticide-free, 87% of fruit samples had pesticide residues below MRL, while 12% of samples exceeded permissible residue levels. All pesticides were in the range 0.002–2.23 mg kg^−1^ and the most frequently detected compounds were fluopyram, imazalil, chlorpyrifos, and indoxacarb [47]. In fruits from the local market in Jordan, 26% of the samples contained pesticides above the MRLs. Furthermore, 10 pesticides in 20 samples exceeded the established MRLs [23]. In fruits from Turkey, 72.2% of the samples showed no pesticide residues, while 10 samples exceeded the maximum residue limits (MRLs) specified in the EU directives. Pyrimethanil and pyriproxyfen were the most frequently detected analytes in the range of 0.05–0.6 mg kg^−1^ [48]. In another study from this market, 60% of the fruits had pesticides below the MRLs [10]. The highest levels of pesticides were detected in apples, cherries, pears, grapes, oranges, and mandarins ranging from 0.01 mg kg^−1^ to 4.16 mg kg^−1^, while 8.4% of the samples exceeded the MRLs [10]. Out of 52 pesticides, residues of 12 pesticides were detected and bifenthrin, fenvalerate, and cypermethrin were frequently detected in a maximum number of fruit and vegetable samples from Turkey. Results showed that 98.8% of tomato, 97.5% of banana, 90% of eggplant, 88.8% of pomegranate, 83.8% of orange, 75% of okra, and 66.3% of green chili samples were below the maximum residue limit (MRL) [10].

Among 744 vegetable samples analyzed in this study, pesticide residues were detected in 44.1% and MRL was exceeded in 54 samples (7.3%) (Figure 2). Cases of MRL exceedances were most often indicated for chlorpyrifos in dill (22 samples) in the range of 120–7900%, followed by linuron in 3 samples (125–365%), tetraconazole in 2 samples (190–4550%), terbuthylazine (115%), and beta-cyfluthrin (700%). Moreover, high MRL exceedances were noticed for dimethoate in green beans (4000%), pirimiphos-methyl in lupine (1600%), and linuron in parsnip (1200%) (Table 3).

Vegetables were characterized by higher variability of detected pesticide residues. Out of 550 pesticides analyzed, 69 were detected. The most frequent group of pesticides in vegetables were also fungicides (33 compounds), followed by insecticides (23), herbicides (11), and acaricides (2). In the group of fungicides, commonly detected were azoxystrobin (8.6%), difenoconazole (7.6%), propamocarb (6.4%), and boscalid (6.3%). The concentration of fungicides in vegetables was in the range of 0.005–7.1 mg kg^−1^, reaching the highest level for difeconazole in dill (Figure 3). Among insecticides, the most frequently determined were chlorpyrifos (7.4%), cypermethrin (2.8%), and acetamiprid (2.5%). Their level varied from 0.005 mg kg^−1^ to 0.8 mg kg^−1^, reaching the greatest content in the case of chlorpyrifos in dill. In the group of herbicides, frequently detected were pendimethalin (10.6%), followed by prosulfocarb (4.4%) and aclonifen (2%) ranging from 0.005 mg kg^−1^ to 0.6 mg kg^−1^ and reaching the highest concentration for aclonifen in dill. Only two acaricides (hexythiazox, 0.3% and fenazaquin, 0.1%) were detected in tomatoes and cucumbers (Figure 3).

According to the European pesticide monitoring program, the pesticides most commonly detected in European fruits and vegetables were cooper compounds, fosetyl, chlorate, hydrogen cyanide, and bromide ion [49]. Similar to this study, cases of MRL exceedances in European fruit and vegetable samples are low (3.7%) [49]. However, scientific studies of pesticide residues in European agricultural crops are very limited, and worldwide research refers mainly to fruits and vegetables from Asia and Africa, where percentages of samples with pesticide residues are more frequent compared to this research. It results from the agricultural practices in countries outside the EU, mainly excessive food production, higher use of pesticides, and application of pesticides at higher concentration. Also, greater temperatures and drought periods in countries outside the Europe increase the survival rate of pests, leading to the need of higher doses of pesticides application [50].

In the study by Tong et al. [51], 36 out of 68 pesticides were detected in Chinese vegetables. The most common substances were acetamiprid, dimethomorph, and propamocarb in the range of 0.0005–3.4 mg kg^−1^. In turn, in vegetables from Sri Lanka, the most frequently detected pesticides were chlorpyrifos, profenofos, tebuconazole, diazinon, and fipronil ranging from 0.004 to 21.98 mg kg^−1^ [52]. In Egypt, the presence of pesticide residues was found in 176 samples of the most popular vegetables collected at markets in the concentration range from 0.01 mg kg^−1^ to 1.4 mg kg^−1^ [53]. The 111 (63.1%) samples were contaminated with pesticide residues, of which 29 samples (16.5%) contained residues higher than the maximum allowable limits (MRLs) [53]. In other studies from Egypt, pesticides were determined in 88% of samples, in which 48% of detected compounds were insecticides and 40% fungicides [54]. The most frequently detected pesticides were chlorpyrifos (25.6%), azoxystrobin (17.4%), and acetamiprid (15.4%) [54]. In vegetables from Bangladesh, commonly detected pesticides were chlorpyrifos, dimethoate, diazinon, and malathion ranging from 0.006 to 1.8 mg kg^−1^ [55]. In 7028 vegetable samples from Shanghai tested from 2018 to 2022, 29.21% of the samples contained pesticide residues, and 0.47% of the samples exceeded the maximum residue limits specified by the national food safety standards in China. Leafy vegetables had the highest detection rate of pesticide residues (32.9%) [51]. Thirty-six of sixty-eight pesticides were detected in vegetables, and the top three pesticides were dimethomorph, propamocarb, and acetamiprid [51]. Of the 52 pesticides detected in fruits from India, 12 were detected, and bifenthrin, fenvalerate, and cypermethrin were frequently detected in the maximum number of samples [30]. The results showed that 98.8% of tomato, 97.5% of banana, 90% of eggplant, 88.8% of pomegranate, 83.8% of orange, 75% of okra, and 66.3% of green chili samples were below the maximum residue limit (MRL) [30].

### 3.3. Multiple Pesticide Residues in Fruit and Vegetable Samples

Multiple pesticide applications during a single growing season, as well as post-harvest (especially during long-term storage or transport), are effective ways to protect fruits and vegetables from pests and diseases, as well as mycotoxins [56]. However, this approach can lead to the accumulation of multiple pesticide residues. Importantly, exposure to multiple pesticides can have synergistic effects on human health, and their cumulative effect leads to an increased spectrum of adverse health effects compared to individual pesticides alone [57].

**Figure 4:**
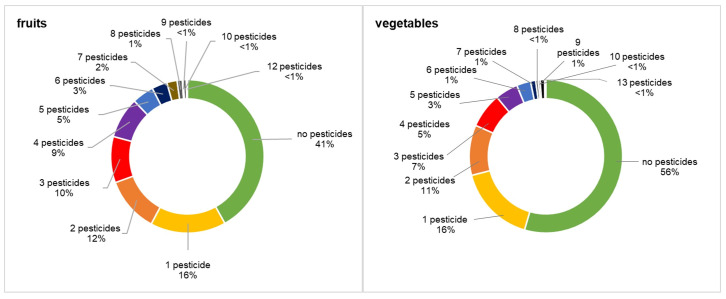
Presence of single and multiple pesticide residues in fruit and vegetable samples.

Of the 370 fruit samples tested, 153 were free of pesticide residues (41%), 59 contained a single compound (16%), and 158 were characterized by the occurrence of multiple residues (42.7%), among which samples with two pesticides were the most common (12%) (Figure 4). On the other hand, one sample of cherry was characterized by 12 residues reaching a total concentration of 1.084 mg kg^−1^. In the case of 744 vegetable samples, 56% were free of pesticides, 16% contained 1 pesticide, while 42.2% of samples were multiresidues, containing from 2 to 13 pesticides (Figure 4). The most frequent among them were two residual samples (11%), followed by three pesticidal (7%), and four residual (5%). The highest number of pesticides (13) was detected in one dill sample reaching a total concentration of 0.562 mg kg^−1^.

According to the European pesticide monitoring program, pesticide multiresidues were detected in 23% of samples, mainly fruits: sweet pepper, grape, strawberry, apple, peach, orange, lemon, pear, lettuce, mandarin, and tomato [49].

In other studies of Polish agricultural crops, 50.4% of fruit samples contained residues of multiple pesticides, most often two compounds (15.3%), while 24.5% of vegetables were characterized by multiple pesticides, commonly by two substances (9.4%) at the total level 0.2–7.6 mg kg^−1^ [17].

In fruits from Egypt, the number of detected pesticide residues ranged from 1 to 20, with the highest diversity found in apples (20), grapes (18), and apricots (14) up to 0.99 mg kg^−1^ of the total concentration. The number of pesticides in each vegetable sample ranged from 1 to 15, with the highest diversity found in cucumbers (15), peppers (12), and zucchini (12) reaching the total level 0.004–0.54 mg kg^−1^ [58]. The most frequently detected compounds were chlorpyrifos and lambda-cyhalothrin [58]. In a study conducted in Uganda, each of the 160 fruit and vegetable samples contained pesticides, and 95.6% of them had at least 2 different residues. In 63.8%, 2 to 9 compounds were detected, while in 25.6% more than 10, which is explained by poor agricultural practices, especially the use of a mixture of pesticides, as well as the uptake of persistent pesticides by plants and spray drift [59]. In Botswana crops, samples with multiple residues accounted for 54%, mainly ranging from 2 to 10 pesticides in one sample [60]. Of nearly 500 fruit and vegetable samples from Turkey, 46% contained more than one residue; however, only 12% contained four or more pesticides [61]. In another Turkish study, out of 2992 fruit and vegetable samples, 11.8% contained two or more residues. The highest proportion of multiresidue samples was recorded for peaches (44.6%), pears (38.4%), cherries (30.0%), grapes (25.8%), and nectarines (25%) [62]. In the case of fruits from China, pesticide residues were detected in samples of strawberries (93.7%), watermelons (82%), melons (88%), peaches (70%), and grapes (100%), and more than 56% of samples had at least two pesticides [63]. Wu et al. [64] found 19 pesticides in garlic from China.

The presence of many pesticide residues is typical for fruits and vegetables that are particularly susceptible to pathogens and that often require multiple, sequential applications of pesticides. This study indicated that pome fruits (including raspberries, strawberries, and blueberries) and dill are the most susceptible to pests. Moreover, problems with the appropriate use of chemical protection in minor crops (especially dill) due to the withdrawal of pesticides in the EU lead to the application of many compounds that are not dedicated to particular crops.

### 3.4. Pesticides Not Approved for Fruits and Vegetables in the EU

According to Regulations 1107/2009 [65] and 540/2011 [66] many pesticides for protecting fruit and vegetables have been withdrawn from the EU market due to their toxic effects on humans, animals, and the environment. This situation caused serious problems with the protection of fruits and vegetables. The lack of effective chemical control of pests contributed to the illegal application of pesticides. The reflection of this tendency in this study was cases of detections of not approved in EU pesticides.

In this scientific investigation, 8 out of 38 pesticides (21%) detected in fruits were not approved for use in the EU. They were found in 11 fruit samples (3%): raspberries, cherries, apples, plums, and blueberries, ranging from 0.006 mg kg^−1^ to 0.42 mg kg^−1^ (Table 4). Carbendazim was the most frequent (1.2%), followed by indoxacarb (0.5%) (Figure 3). Within the particular group of fruit assortments, the highest percentages of pesticides were determined for thiacloprid and indoxacarb in apples (20% and 10%, respectively) and carbendazim in cherries (9.4%) (Table 4). In the case of vegetables, 24 not approved pesticides out of 69 compounds (35%) were determined in the range of 0.006–0.8 mg kg^−1^. They were detected in 93 vegetable samples (12.5%), mainly dill. Chlorpyrifos was the most commonly detected pesticide in vegetables (7.4%). Among vegetable assortments, the highest percentages of pesticides were indicated for mepaniprim in parsley (100%), followed by 2,6-dichlorobenzamide in lettuce (50%), chlorpyrifos in dill and carrot (43.5% and 33%, respectively), and dimethomorph in tomato and potato (25.9% and 25%, respectively) (Table 4).

### 3.5. Acute Risk Assessment of Children and Adults Resulting from the Consumption of Fruits and Vegetables with Residues Exceeding MRL

The acute exposure to pesticide residues was assessed according to EFSA methodology [67]. This model is a universal and international tool for all European countries with built-in consumption data of the most critical European children’s and adults’ diets and allows for comparison with other researchers.

In this study, acute risk assessment was performed for 41 fruit/vegetable samples exceeding the established safety level (MRL). It is important to mention that most of these pesticides are not approved in the European Union due to their toxicological effects on human health including nervous, cardiovascular, and respiratory disorders (Table 5). For example, chlorpyrifos, dimethoate, and omethoate are acetylcholinesterase inhibitors [63]. Flutriafol is an endocrine disruptor, while beta-cyfluthrin, linuron, and propiconazole are reproductive disruptors [68,69,70]. Nevertheless, they are still widely used in agriculture practice in broccoli, brussels sprouts, cauliflower, head cabbage, and potatoes. Moreover, among pesticides exceeding the MRL, indoxacarb cause neurological effects and skin sensitization, while dodine, pirimiphos-methyl, hexythiazox, and terbuthylazine are eye, skin, and respiratory tract irritants [71,72,73,74].

The short-term intakes were considered for the most critical subpopulations taking into account different input values for calculation: in the case of IESTI, it was the pesticide residue level above MRL, and in the case of IESTInew, this value was the MRL limit. The results for each subpopulation of adults and children are summarized in Table 5.

The calculated acute exposures were below the health-based acute reference values in all tested samples under the 2-year cycle with one exception. The maximum acute intake value IESTI = 156.5% (Case 2b) was estimated for linuron in the case of British infants resulting from consumption of parsnip in diet (UK infant, bw = 8.7 kg, LP = 44.9 g person^−1^).

Exposure of children consuming fruit and vegetables containing pesticide residues exceeding MRL ranged from 0.1% ARfD (lentil/pirimiphos methyl) to 156.5% ARfD (parsnips/linuron) in case of IESTI and to 12.3% ARfD (apples/indoxacarb) in case of IESTI_new_. Acute intakes in the subpopulation of adults were lower than in children and IESTI were in the range of 0.0% ARfD (lentil/pirimiphos methyl)–60.9% ARfD (parsnip/linuron), while IESTI_new_ up to 6.4% ARfD (tomato/hexythiazox).

For one sample, namely, parsnip containing 0.13 mg kg^−1^ of linuron (MRL= 0.01 mg kg^−1^), the estimated value of exposure exceeded 100% ARfD (IESTI= 156.5% ARfD), which might pose a risk for consumers. Therefore, in this case, the new threshold residue level was established TRL = 0.083 mg kg^−1^. It is important to note that the proposed EFSA assessment methodology overestimated the intakes.

It is important to note that fruits and vegetables in this study were fresh, without any processing treatment. However, it should be considered that fruit and vegetables usually undergo pre-treatment before consumption (e.g., washing, peeling, homogenization) or are processed using high or low temperatures (e.g., boiling, cooking, freeze-drying). Household or industrial processing can reduce pesticide residue levels and thus minimize health exposure [17,75,76,77,78].

Based on the 2022 European Union pesticide monitoring in food carried out with 193 pesticides [49], estimated dietary exposure to pesticide residues was very low for most of the EU subpopulation groups. The estimated intake exposure exceeded the health-based guidance value (ARfD) by 1.7% for the specific pesticide/product combinations, e.g., phosmet, imazalil, lambda-cyhalothrin, cypermethrin, acetamiprid in grapefruits, pears, peaches, oranges, and apples.

## 4. Conclusions

This comprehensive study underscores a critical issue within the monitoring of pesticide residues in fruits and vegetables. The use of a three-component clean-up mixture consisting of GCB, PSA, and MgSO_4_ resulted in minimizing the matrix effect and obtaining good validation parameters. Fruits were characterized by higher contamination with pesticides (58.6%) and a lower number of cases with MRL exceedances (1.9%) than vegetables (44.1% and 7.3%, respectively). Moreover, a higher number of pesticides not approved in the EU was found in vegetables (24 compounds) than in fruits (8 compounds). The most frequently detected groups of pesticides in fruits and vegetables were fungicides and insecticides, which confirms that they are very sensitive to fungal diseases and pests. Herbicides, which are used to control monocotyledonous and dicotyledonous weeds that compete with crops for nutrients, water, and sunlight, were often detected in vegetable samples. Toxicological health risk assessment provides a more realistic consumer exposure to pesticide residues in diet and revealed that out of 1114 samples of examined fruits and vegetables, the acute health risk exceeding 100% of the acute reference dose was calculated in only one case (parsnip/linuron/children), indicating that Polish crops collected for governmental official control are safe. Based on these results, it is recommended to only use pesticides approved in the EU and follow the rules of the grace period in agricultural practice to avoid the presence of pesticides in fruits and vegetables. Moreover, continuous monitoring of pesticide residues in all fruit and vegetable products is highly recommended in order to reduce the risk to consumer health by limiting the number of samples exceeding MRLs and containing substances not approved for use in Europe.

## Figures and Tables

**Table 2:** Population data for the assessment of acute health risk of children and adults in relation to raw fruits and vegetables.

	Assortment	Case	Children				Adults		
			MS Critical Diet	Body Weight (kg)		Large Portion (g person^−1^)	MS Critical Diet	Body Weight (kg)	Large Portion (g person^−1^)
Fruits	Apple	2a	NL toddler	10.2		209.4	FR adult	66.4	664.0
	Plum	2a	IE child	20.0		170.6	DE women 14–50	67.5	879.1
	Raspberry	1	IE child	20.0		184.7	FI men	84.7	456.8
Vegetables	Beans	1	NL toddler	10.2		116.6	NL general population	65.8	507.6
	Dill	1	NL toddler	10.2		4.9	NL general population	65.8	21.5
	Kale	2b	DE child	16.15		142.1	DE general population	76.4	294.1
	Lentil	3	UK 11–14 years	48.0		321.5	FR adult	66.4	408.4
	Parsnip	2b	UK infant	8.7		44.9	UK vegetarian	66.7	188.0
	Tomato	2a	BE toddlers	17.8		180.0	LT adult	70.0	450.0
Acute risk assessment equation for commodities with different unit weight (U): Case 1 for crops with U ≤ 25 g IESTI = [LP × HR × CF]/bw Case 2 for crops U > 25 g Case 2a where U < LP IESTI = [U × HR × CF + (LP − U) × HR × CF]/bw Case 2b where U > LP IESTI = [LP × HR × CF × VF]/bw Case 3 for food products that are usually bulked or blended before they are consumed (e.g., cereals, pulses). IESTI = [LP × HR × CF]/bw					Parameters: IESTI—International Estimated Short-Term Intake; LP—Large Portion (97.5th percentile of eaters) (g kg^−1^ bw); HR—Highest Residue according to residue definition for enforcement in the composite sample (mg kg^−1^); CF—Conversion Factor residue definition enforcement to residue definition risk assessment. VF—Variability Factor; VF = 1 when U < 25 g; VF = 7 when U between 25 and 250 g; VF = 5 when U above 250g; bw—body weight for the subgroup of the population related to the LP or mean consumption (kg).				

BE—Belgium; DE—Germany; FI—Finland; FR—France; IE—Irland; LT—Lithuania; NL—Netherlands; UK—United Kingdom.

**Table 3:** Maximum residue level (MRL) exceedances in fruit and vegetable samples.

No.	Assortment	Pesticide	Toxicological Effects	Number of Samples Exceeding MRL	Detected Concentration (mg kg^−1^)	MRL (mg kg^−1^)	MRL Exceeding (%)
Fruits							
1	Apple	Indoxacarb	• Neurotoxicant • Skin sensitizer	1	0.026	0.01	160
2	Raspberry	Flutriafol	• Respiratory tract irritant • Eye irritant • Endocrine disruptor	1	0.030	0.01	200
3	Plum	Dodine	• Eye irritant • Skin irritant	1	0.052	0.01	420
Vegetables							
1	Green bean	Dimethoate	• Acetyl cholinesterase inhibitor • Eye irritant	1	0.410	0.01	4000
		Metalaxyl	-	1	0.110	0.02	450
		Omethoate	• Acetyl cholinesterase inhibitor • Endocrine disruptor • Neurotoxicant • Skin irritant • Skin sensitizer	1	0.098	0.01	880
2	Kale	Flonicamid	-	1	0.091	0.03	203
3	Dill	Beta-cyfluthrin	• Reproduction/development effects • Respiratory tract irritant • Neurotoxicant	1	0.160	0.02	700
		Chlorpyrifos	• Reproduction/development effects • Acetyl cholinesterase inhibitor • Endocrine disruptor • Neurotoxicant	22	0.022–0.79	0.01	120–7900
		Linuron	• Reproduction/development effects • Eye irritant • Skin irritant	3	0.045–0.093	0.02	125–365
		Terbuthylazine	• Respiratory tract irritant • Eye irritant • Skin sensitizer	1	0.043	0.02	115
		Tetraconazole	-	2	0.058–0.930	0.02	190–4550
4	Lupine	Pirimiphos-methyl	• Respiratory tract irritant • Eye irritant • Acetyl cholinesterase inhibitor • Skin irritant	1	0.170	0.01	1600
5	Parsnip	Chlorpyrifos	• Reproduction/development effects • Acetyl cholinesterase inhibitor • Endocrine disruptor • Neurotoxicant	1	0.031	0.01	210
		Linuron	• Reproduction/development effects • Eye irritant • Skin irritant	1	0.130	0.01	1200
		Propiconazole	• Reproduction/development effects • Respiratory tract irritant • Endocrine disruptor	1	0.024	0.01	140
6	Tomato	Hexythiazox	• Respiratory tract irritant • Eye irritant • Skin irritant	1	0.280	0.1	180

**Table 4:** Pesticides not approved in the EU that were detected in this study.

Assortment	Pesticide ^a^	Concentration Range (mg kg^−1^)	Number of Samples Tested	Number of Samples with Not Approved Pesticide in the EU	% of Samples Within Assortment with Not Approved Pesticides	% of Fruit/Vegetable Samples with Not Approved Pesticides
FRUITS						
Rasberry	Alpha-cypermethrin (I)	0.045	36	1	2.8	0.3
	Flutriafol (F)	0.03		1	2.8	0.3
	Carbendazim (F)	0.11		1	2.8	0.3
	Chlorpyrifos (I)	0.014		1	2.8	0.3
Cherry	Fenpyrazamine (F)	0.42	32	1	3.1	0.3
	Carbendazim (F)	0.006–0.01		3	9.4	0.8
Apple	Indoxacarb (I)	0.007	10	1	10.0	0.3
	Thiacloprid (I)	0.014–0.026		2	20.0	0.5
Plum	Indoxacarb (I)	0.006	39	1	2.6	0.3
	Carbendazim (F)	0.018–0.042		2	5.1	0.5
	Thiophanate-methyl (F)	0.017		1	2.6	0.3
Blueberry	Carbendazim (F)	0.007	48	1	2.1	0.3
VEGETABLES						
Dill	2,6-dichlorobenzamide (H)	0.01–0.8	138	24	17.4	3.2
	Beta-cyfluthrin (I)	0.16		1	0.7	0.1
	Chlorpyrifos (I)	0.006–0.8		60	43.5	8.0
	Chlorothalonil (F)	0.009		1	0.7	0.1
	Dimethomorph (F)	0.006–0.009		2	1.4	0.3
	Carbendazim (F)	0.007–0.039		3	2.2	0.4
	Linuron (H)	0.007–0.093		7	5.1	0.9
	Metalaxyl (F)	0.074		1	0.7	0.1
	Metolachlor (H)	0.007		1	0.7	0.1
	Metribuzin (H)	0.006–0.048		10	7.2	1.3
	Thiacloprid (I)	0.009		1	0.7	0.1
	Thiophanate-methyl (F)	0.015		1	0.7	0.1
Tomato	2,6-dichlorobenzamide (H)	0.012	54	1	1.9	0.1
	Chlorpyrifos (I)	0.006		1	1.9	0.1
	Dimethomorph (F)	0.006–0.5		14	25.9	1.9
	Dithiocarbamates (F)	0.05–0.06		3	5.6	0.4
	Famoxadone (F)	0.009		1	1.9	0.1
	Fenpyrazamine (F)	0.021		1	1.9	0.1
	Imidacloprid (I)	0.015		1	1.9	0.1
	Carbendazim (F)	0.011		1	1.9	0.1
	Thiophanate-methyl (F)	0.11		1	1.9	0.1
Cucumber	2,6-dichlorobenzamide (H)	0.007–0.016	23	4	17.4	0.5
	Chlorpyrifos (I)	0.007		1	4.3	0.1
	Chlorothalonil (F)	0.018		1	4.3	0.1
	Dimethomorph (F)	0.006–0.027		5	21.7	0.7
Broad bean	Thiacloprid (I)	0.014	10	1	10.0	0.1
Horseradish	p,p’ DDE (I)	0.029	15	1	6.7	0.1
Green bean	Imidacloprid (I)	0.014	24	1	4.2	0.1
	2,6-dichlorobenzamide (H)	0.016		1	4.2	0.1
	Dimethoate (I)	0.41		1	4.2	0.1
	Omethoate (I)	0.098		1	4.2	0.1
Kale	Metribuzin (H)	0.009	10	1	10.0	0.1
Carrot	Chlorpyrifos (I)	0.011	3	1	33.0	0.1
Parsnip	Linuron (H)	0.015–0.13	12	2	16.7	0.3
	Chlorpyrifos (I)	0.031		1	8.3	0.1
	Propiconazole (F)	0.024		1	8.3	0.1
	Thiacloprid (I)	0.015		1	8.3	0.1
Parsley	Mepanipirim (F)	0.009	1	1	100.0	0.1
Leek	Pirimiphos-methyl (I)	0.015	27	1	3.7	0.1
	Spinetoram (I)	0.021		1	3.7	0.1
	Imidacloprid (I)	0.015		1	3.7	0.1
Lettuce	2,6-dichlorobenzamide (H)	0.015–0.027	6	3	50.0	0.4
	Cyfluthrin (I)	0.33		1	16.7	0.1
	Metalaxyl (F)	0.18		1	16.7	0.1
Potato	Dimethomorph (F)	0.007–0.008	8	2	25.0	0.3

^a^ (F)—fungicide; (I)—insecticide; (H)—herbicide.

**Table 5:** Acute health risk assessment for children and adults consuming fruits and vegetables containing pesticide residues exceeding MRL: IESTI (input value: residue level > MRL) and IESTI _new_ (input value: MRL).

Assortment	MRL (mg kg^−1^)	Residue Level (mg kg^−1^)	Pesticide	ADI (mg kg^−1^ bw day^−1^)	ARfD (mg kg^−1^ bw)	Case	Children IESTI (%ARfD)	Children IESTI_new_ (%ARfD)	Adults IESTI (%ARfD)	Adults IESTI_new_ (%ARfD)
FRUITS										
Apples	0.01	0.026	Indoxacarb ^a^	0.005	0.005	2a	56.0	12.3	14.6	6.0
Plum	0.01	0.052	Dodine	0.1	0.1	2a	2.2	0.3	0.9	0.4
Raspberry	0.01	0.03	Flutriafol ^a^	0.01	0.05	1	0.6	0.2	0.3	0.1
VEGETABLES										
Beans	0.01	0.41	Dimethoate ^a^	0.001	0.01	1	46.9	1.1	31.6	0.8
	0.02	0.11	Metalaxyl	0.08	0.5	1	0.3	0.1	0.2	0.0
	0.01	0.098	Omethoate ^a^	0.0004	0.002	1	56.0	5.7	37.8	3.9
Dill	0.02	0.16	Beta-cyfluthrin ^a^	0.01	0.01	1	0.7	0.1	0.5	0.1
	0.01	0.022–0.80	Chlorpyrifos ^a^	0.001	0.005	1	0.2–7.7	0.1	0.1–5.2	0.1
	0.02	0.045–0.093	Linuron ^a^	0.003	0.003	1	0.7–1.5	0.3	0.5–1.0	0.2
	0.02	0.043	Terbuthylazine	0.004	0.008	1	0.3	0.1	0.2	0.1
	0.02	0.058–0.093	Tetraconazole	0.004	0.05	1	0.1–0.9	0.0	0.0–0.6	0.0
Kale	0.03	0.091	Flonicamid	0.025	0.025	2b	16.0	3.2	7.0	1.4
Lentil	0.01	0.01	Pirimiphos methyl	0.004	0.15	3	0.1	0.1	0.0	0.0
Parsnip	0.01	0.031	Chlorpyrifos ^a^	0.001	0.005	2b	22.4	3.1	8.7	1.7
	0.01	0.13	Linuron ^a^	0.003	0.003	2b	**156.5**	5.2	60.9	2.8
	0.01	0.024	Propiconazole ^a^	0.04	0.1	2b	0.9	0.2	0.3	0.1
Tomato	0.1	0.28	Hexythiazox	0.03	0.03	2a	54.3	10.1	14.8	6.4

^a^ pesticide not approved in the EU. The bold value indicates possible acute health risk.

## Data Availability

The original contributions presented in this study are included in the article/Supplementary Materials. Further inquiries can be directed to the corresponding authors.

## Supplementary Materials

The following supporting information can be downloaded at: https://www.mdpi.com/article/10.3390/foods14142528/s1, Table S1: Chromatographic conditions and the triple quadrupole system parameters of LC/GC/MS/MS instruments. Table S2: Acquisition parameters for the compounds and internal standards (ISs) analyzed by LC/GC/MS/MS. Table S3: Validation parameters: recovery (%), relative standard deviation (RSD%), correlation coefficient (R2), limit of quantification (LOQ), matrix effect (ME%), and expanded uncertainty (U%) of 550 pesticides in carrots, dill, green beans, leeks, raspberries, cherries, and tomatoes (excel file). Table S4: Results of participation in proficiency testing in fruits and vegetables.
