# Supplementary material for: Evaluation of Broad-Spectrum Pesticides Based on Unified Multi-Analytical Procedure in Fruits and Vegetables for Acute Health Risk Assessment
Source: Foods. 2025 Jul 18;14(14):2528. doi: 10.3390/foods14142528 (PMC12294294; doi:10.3390/foods14142528)
Supplement: Supplementary file 1 [file foods-14-02528-s001.zip › supplementary material.pdf]

Evaluation of a broad-spectrum pesticides based on unified multi analytical procedures in fruits and vegetables for acute health risk assessment

Bożena Łozowicka[1], Piotr Kaczyński[1], Magdalena Jankowska[1], Ewa Rutkowska[1], Piotr Iwaniuk[1,*], Rafał Konecki[1], Weronika Rogowska[1], Aida Zhagyparova[2], Damira Absatarova[3,*], Stanisław Łuniewski[4], Marcin Pietkun[5], Izabela Hrynko[1]

[1] Institute of Plant Protection – National Research Institute, Chełmońskiego 22 St., 15-195 Białystok, Poland

[2] L.N. Gumilyov Eurasian National University, Satbayev 2 St., 010008 Astana, Kazakhstan

[3] Zhetysu University, Zhansugurov 187A St., 040009 Taldykorgan, Kazakhstan

[4] Eastern European University of Applied Sciences in Białystok, Ciepła 40 St., 15-472 Białystok, Poland

[5] Hydratec, Radziwonika 12 St., 15-166 Białystok, Poland

* Correspondence: p.iwaniuk@iorpib.poznan.pl (P. Iwaniuk), mikalok.kz@mail.ru (D. Absatarova).

Caption:

Table S1. Chromatographic conditions and the triple quadrupole system parameters of LC/GC/MS/MS instruments.

Table S2. Acquisition parameters for the compounds and internal standards (ISs) analyzed by LC/GC/MS/MS.

Table S3. Validation parameters: recovery (%), relative standard deviation (RSD%), correlation coefficient (R2), limit of quantification (LOQ), matrix effect (ME%), and expanded uncertainty (U%) of 550 pesticides in carrots, dill, green beans, leeks, raspberries, cherries, and tomatoes (excel file).

Table S4. Results of participation in proficiency testing in fruits and vegetables.

Table S1. Chromatographic conditions and triple quadrupole system parameters of LC/GC/MS/MS instruments.

System LC	Eksigent Ultra LC-100	System GC	Agilent 7890B
Column	KINETEX C18 2.6 μm, 2.1 x 100 mm	Column	HP-5MS<br>30 m × 0.25 mm ID and 0.25 μm
Column temperature	40 °C	Column temperature	70 °C
Injection volume	10 μl	Injection mode	Splitless
Mobile phase	A: water with 0.5% formic acid and 2mM ammonium formate<br>B: methanol with 0.5% formic acid and 2 mM ammonium formate	Injection volume<br>Injection temperature	2 μl<br>270 °C
Gradient	0–0.5 min 1% B→0.5–5 min 1–90% B→5-7 min 90% B→7–8 min 90–1% B→8–10 min 1% B	Oven temperature	70 °C (2 min hold) → 150 °C at 25 °C/min → 200 °C at 3 °C/min → 280 °C at 8 °C/min (10 min hold).
Flow rate	0.50 mL/min	Carrier gas (flow)	Helium (2.1 mL/min)
Total running time	10 min	Total running time	42.88 min
System MS/MS	6500 QTRAP	System MS/MS	Agilent 7000B
Ionisation mode	Electrospray positive ion mode	Ionisation mode	Electron ionisation source (-70 eV)
Capillary voltage	5000 V	Transfer line temperature	280 °C
Turbo heaters	400 °C	Ion source temperature	300 °C
Nebulizer gas (pressure)	Nitrogen (60 psi)	Quadrupoles temperature	180 °C, 180 °C
Auxiliary gas (pressure)	Nitrogen (50 psi)	Collision gases (flow)	Helium (2.25 mL/min)
Curtain gas (pressure)	Nitrogen (30 psi)		Nitrogen (1.5 mL/min)
Software	Analyst version 1.7.2	MassHunter version B.06.00	

Table S2. Acquisition parameters for the compounds and internal standards (ISs) analyzed by LC/GC/MS/MS.

a) LC/MS/MS

No.	Compound	Precursor ion (m/z)	Quantification			Confirmation			DP(V)	EP(V)
			Product ion (m/z)	CE (V)	CXP (V)	Product ion (m/z)	CE (V)	CXP (V)		
1.	3-hydroxycarbofuran	238.1	181.1	17	10	163	21	10	46	10
2.	8,9-Z-avermectin B1a	890.5	305.1	23	14	567.3	23	14	20	10
3.	Acetamiprid	223	125.9	27	6	99	51	5	80	10
4.	Acibenzolar-S-methyl	211	136	39	8	91	28	11	96	10
5.	Aclonifen	265.1	248	25	14	182.1	39	10	61	10
6.	Alachlor	271.1	162.1	20	10	238.1	11	10	28	10
7.	Aldicarb	208.1	116.1	11	8	89	21	14	26	10
8.	Aldicarb sulfone	223.1	148	13	8	86	23	14	36	10
9.	Aldicarb sulfoxide	207	132	11	8	88.9	21	10	36	10
10.	Allethrin	303.2	123	25	6	91	55	4	66	10
11.	Ametoctradin	276.233	149	51	8	176.1	53	10	96	10
12.	Ametryn	228.1	186	25	10	96	33	14	86	10
13.	Amidosulfuron	370	261	19	14	218	33	12	25	10
14.	Aminocarb	209.1	137.1	33	8	152.1	19	10	56	10
15.	Amisulbrom	466	227	5	25	148.1	5	12	81	10
16.	Anilazine	275	153	37	12	178	35	10	76	10
17.	Anthraquinone	209.2	152	45	12	153.1	33	8	76	10
18.	Atrazine desisopropyl	174.1	104.1	33	18	132.1	25	8	61	10
19.	Avermectin B1a	890.4	305.1	33	16	567.2	21	26	62	10
20.	Avermectin B1b	876.5	291	35	10	145	43	6	91	10
21.	Azadirachtin	703.219	567.1	21	8	585.1	19	8	96	10
22.	Azamethiphos	325	112.1	51	2	138.9	37	8	86	10
23.	Beflubutamid	356.2	91.1	53	6	65	105	10	66	10
24.	Bendiocarb	224.11	167.1	13	10	109	23	8	36	10
25.	Benfuracarb	411.2	195.1	28	10	89.9	21	10	66	10
26.	Benomyl	291.2	160.1	39	12	132.1	69	8	41	10
27.	Bensulfuron-methyl	411	149	27	8	118.9	57	12	101	10

No.	Compound	Precursor ion (m/z)	Quantification			Confirmation			DP(V)	EP(V)
			Product ion (m/z)	CE (V)	CXP (V)	Product ion (m/z)	CE (V)	CXP (V)		
28.	Benthiavalicarb-isopropyl	382.1	180	41	10	116	27	6	91	10
29.	Benzoximate	364	199	13	10	104.9	35	12	56	10
30.	Bifenazate	301.1	170	27	10	151.9	55	8	76	10
31.	Bifenox	341.9	309.9	10	10	188.9	22	10	16	10
32.	Bixafen	414.2	393.9	21	24	266	33	14	76	10
33.	Bromacil	261	204.9	19	12	187.9	37	10	36	10
34.	Butafenacil	492.1	331	33	18	180	57	10	46	10
35.	Butocarboxim	207	132.1	9	8	74.9	19	12	31	10
36.	Butocarboxim sulfoxide	223.1	106	11	6	166.1	15	8	40	10
37.	Buturon	237	84	21	14	125.9	39	6	91	10
38.	BYI08330-enol	302.1	216.1	28	13	268.1	24	14	46	10
39.	BYI08330-enol-glucoside	464.2	302.1	12	13	270.1	48	15	22	10
40.	BYI08330-ketohydroxy	318.1	300.2	18	12	270.1	18	10	24	10
41.	BYI08330-monohydroxy	304.1	254.1	20	10	272.1	28	11	36	10
42.	Carbendazim	192	160.1	27	10	132.1	43	8	71	10
43.	Carbetamide	237.1	192.1	13	12	118.1	17	6	36	10
44.	Carfentrazone-ethyl	412	345.9	33	18	365.9	25	20	126	10
45.	Chlorbromuron	292.9	204	28	10	182	25	9	51	10
46.	Chlorfluazuron	540	382.9	31	20	158	25	10	81	10
47.	Chloridazon	222	104	31	12	92	33	14	11	10
48.	Chlorotoluron	213.1	72	21	10	46	37	12	76	10
49.	Chloroxuron	291	72	23	8	218	33	11	106	10
50.	Chlorsulfuron	358	167	23	10	141.1	23	6	25	10
51.	Chromafenozide	395.2	175	21	10	339.1	11	19	40	10
52.	Cinosulfuron	414	183	23	10	215	22	11	76	10
53.	Clethodim	360.1	164.1	29	10	268.1	17	16	61	10
54.	Clodinafop-propargyl	350	266	21	14	90.9	35	14	101	10
55.	Clofentezine	303	138	19	8	101.9	49	12	51	10
56.	Cloquintocet mexyl	336.2	237.9	21	20	191.9	37	30	86	10

No.	Compound	Precursor ion (m/z)	Quantification			Confirmation			DP(V)	EP(V)
			Product ion (m/z)	CE (V)	CXP (V)	Product ion (m/z)	CE (V)	CXP (V)		
57.	Clothianidin	250	169	19	10	132	21	6	6	10
58.	Coumaphos	363.004	227	37	12	306.9	25	18	81	10
59.	Crimidin	172	106.9	35	14	95.1	31	8	91	10
60.	Cyanazine	241.1	214.1	25	12	104	43	18	61	10
61.	Cyantraniliprole	472.7	283.9	14	10	442.0	17	10	32	10
62.	Cyazofamid	325.1	108	19	8	261	13	14	16	10
63.	Cycloxydim	326.2	280.1	19	14	180	27	10	91	10
64.	Cycluron	199.1	89	21	10	72	29	10	71	10
65.	Cymoxanil	199	128	13	4	111	25	4	81	10
66.	Cyprazine	228.1	186	25	10	108	33	12	96	10
67.	Dazomet	163.1	107.2	11	6	89.1	15	14	51	10
68.	Demeton-S	259	88.9	21	14	61	49	10	21	10
69.	Demeton-S-methyl	231	88.9	17	12	61	41	10	21	10
70.	Demeton-S-methyl-sulfone	263	169	21	12	108.9	37	14	76	10
71.	Desmedipham	318.1	182.1	19	10	136	37	6	16	10
72.	Diafenthiuron	385.1	329.1	27	16	278.1	43	14	121	10
73.	Diclobutrazol	328	69.9	58	8	159	48	8	85	10
74.	Didecyldimethylammonium chloride (DDAC)	326.2	186.2	39	12	41.1	93	6	61	10
75.	Diethofencarb	268.1	226.1	13	12	180	25	10	26	10
76.	Difenoxuron	287	123	25	8	71.9	23	10	121	10
77.	Diflubenzuron	311.1	158.1	19	10	141	47	8	61	10
78.	N,N-dimethyl-N'-p-tolysulphamide (DMST)	215.2	106	19	8	77.1	55	14	41	10
79.	Dimefuron	339	166.9	29	10	256	23	14	141	10
80.	Dimethylaminosulfanilide (DMSA)	201.2	137.2	13	24	91.9	27	6	46	10
81.	Dinotefuran	203	157.1	11	10	113.1	17	8	36	10
82.	Dioxacarb	224	167.1	13	10	123	23	6	41	10
83.	Disulfoton	275	89	20	10	61	46	10	56	10
84.	Disulfoton-sulfon	307	96.8	41	12	171	16	9	66	10
85.	Disulfoton sulfoxide	291.1	185	19	12	212.9	15	12	46	10

No.	Compound	Precursor ion (m/z)	Quantification			Confirmation			DP(V)	EP(V)
			Product ion (m/z)	CE (V)	CXP (V)	Product ion (m/z)	CE (V)	CXP (V)		
86.	Diuron	233	72	23	8	46.1	39	12	86	10
87.	Dodine	228.3	57	41	10	186.2	27	10	91	10
88.	Doramectin	916.4	331.1	33	16	593.2	21	28	81	10
89.	Emamectin B1a	886.4	158.1	41	10	126	83	22	121	10
90.	Emamectin B1b	872.4	158.1	43	8	126.2	79	16	106	10
91.	Eprinomectin B1a	914.4	186.1	23	4	112	95	6	160	10
92.	Ethametsulfuron-methyl	411.2	196.1	15	10	168.1	30	10	46	10
93.	Ethiofencarb	226.1	107.1	23	8	164.1	11	10	41	10
94.	Ethiofencarb-sulfone	258	107.1	21	8	200.9	11	10	51	10
95.	Ethiofencarb-sulfoxide	242	106.9	23	12	185	13	8	41	10
96.	Ethiprole	397	350.8	29	18	254.9	47	14	116	10
97.	Ethirimol	210.1	140.1	29	8	97.9	35	12	116	10
98.	Ethoxyquin	218.2	148.0	31	12	174.1	41	14	61	10
99.	Ethoxysulfuron	399	261	25	14	218	35	14	56	10
100	Fenamiphos sulfone	336.1	265.9	29	14	308	23	18	81	10
101	Fenamiphos sulfoxide	320.2	171	33	10	233	35	12	86	10
102	Fenbutatin oxide	519.1	90.9	103	14	463.1	33	22	171	10
103	Fenchlorazole-ethyl	403.9	357.8	31	18	375.7	21	20	116	10
104	Fenfuram	202	109	27	8	120	21	6	91	10
105	Fenhexamid	302.1	97.1	31	6	55	57	8	86	10
106	Fenobucarb	208.1	95.1	21	8	152.1	11	10	36	10
107	Fenpicoxamid	615	124,1	74	10	239	22	10	35	10
108	Fenpyroximate	422.1	366.1	23	18	135	41	8	116	10
109	Fensulfothion sulfone	325	268.9	21	14	296.9	15	18	101	10
110	Fenthion sulfone	311.1	125	29	8	278.8	27	16	76	10
111	Fenthion sulfoxide	295.1	279.7	25	16	108.9	45	18	76	10
112	Fenthion oxon sulfone	295	104.1	35	6	217.1	27	12	96	10
113	Fenthion oxon sulfoxide	279.1	104.1	39	18	264.1	27	16	56	10
114	Fenthion oxon	263.1	216	33	14	231	23	14	76	10

No.	Compound	Precursor ion (m/z)	Quantification			Confirmation			DP(V)	EP(V)
			Product ion (m/z)	CE (V)	CXP (V)	Product ion (m/z)	CE (V)	CXP (V)		
115	Fenuron	165.1	72	21	10	46.1	19	8	91	10
116	Flazasulfuron	408	182	25	10	227	28	17	66	10
117	Florasulam	359.9	128.9	20	20	81.6	20	20	50	10
118	Florpyrauxifen-benzyl	439	91	63	5	65	113	10	30	10
119	Fluazinam	463	451.9	30	28	397.9	21	26	30	10
120	Fluazuron	506	158	27	10	141	67	8	126	10
121	Flubendiamid	700	408	19	20	273.9	47	14	50	10
122	Fluconazole	307.1	238,2	18	14	169	34	9	80	10
123	Fluensulfone	292	166	25	10	109	25	10	40	10
124	Flufenoxuron	489	158	25	10	141	71	8	111	10
125	Flumethrin	510.2	239	29	14	267	17	16	91	10
126	Flumioxazin	355	327.2	20	18	299	38	16	136	10
127	Fluometuron	233.1	72.1	23	8	145.1	47	8	86	10
128	Fluopyram	397	173	39	12	145	43	14	54	10
129	Flupyradifurone	288.9	126	5	33	245	5	23	76	10
130	Fluoroglycofene-ethyl	465	343.8	19	18	222.9	45	12	51	10
131	Fluxapyroxad	382.083	342.1	29	20	362.2	21	4	71	10
132	Fluoxastrobin	459	427	25	22	188	45	10	101	10
133	Fluridone	330	310	39	16	259	63	14	161	10
134	Fluthiacet-methyl	404	273.9	39	16	215	53	12	151	10
135	FM-6-1	294.944	215	31	12	43.2	53	2	71	10
136	Foramsulfuron	453	182.2	35	12	272	21	16	71	10
137	Forchlorfenuron	248	129	23	6	93	47	10	36	10
138	Formetanate	222	165.1	21	10	120.1	35	7	16	10
139	Furalaxyl	302.1	242.1	21	12	95	33	8	66	10
140	Furathiocarb	383.1	195	25	12	252	17	14	86	10
141	Halauxifen-methyl	346	286	5	22	251	5	32	30	10
142	Halofenozide	331	105	23	6	275	15	12	60	10
143	Halosulfuron-methyl	435	182	31	10	83.1	79	4	61	10

No.	Compound	Precursor ion (m/z)	Quantification			Confirmation			DP(V)	EP(V)
			Product ion (m/z)	CE (V)	CXP (V)	Product ion (m/z)	CE (V)	CXP (V)		
144	Haloxyfop-ethoxyethyl	434	315.9	27	16	90.9	45	14	106	10
145	Hexazinone	253.1	171	23	10	71	41	12	66	10
146	Hexaflumuron	461	158.1	23	10	141	63	8	111	10
147	Hydramethylnon	495.1	323.1	43	18	151	95	8	211	10
148	Imazamox	306.2	261.1	31	14	193.1	37	12	66	10
149	Imidacloprid	256	209.1	21	12	175.1	27	10	80	10
150	Iodosulfuron-methyl	507.9	167	25	10	141	40	8	71	10
151	Isofetamid	360,1	125	15	10	182	10	10	20	10
152	Isoproturon	207.1	72	23	10	46.1	37	10	91	10
153	Isopyrazam	360.116	320.1	29	16	340.1	23	4	81	10
154	Isoxaben	333.1	165	25	10	106.9	81	12	71	10
155	Isoxadifen-ethyl	313.2	232.1	27	12	204.2	39	12	34	10
156	Isoxaflutole	377.1	69	19	14	251	27	14	55	10
157	Isoxaflutole diketonitrile	377	250.9	33	14	69	31	2	51	10
158	Ivermectin	892.4	307.1	32	14	569.3	21	26	94	10
159	Linuron	249	160	25	10	182	21	10	61	10
160	Lufenuron	511	158.1	25	10	140.9	73	8	111	10
161	Mandestrobin	314.4	192	5	7	160	5	17	20	10
162	Mefenacet	299.1	148	19	8	120.1	35	8	51	10
163	Mefenpyr-diethyl	373.1	326.9	23	16	160.1	47	10	70	10
164	Mefentrifluconazole	397.8	70	25	10	181.9	30	10	20	10
165	Mendipropamid	412.1	328	21	18	356	15	18	96	10
166	Mepanipyrim	224	106	33	18	66	59	10	131	10
167	Mepronil	270.1	119.1	31	8	228	20	12	91	10
168	Mesosulfuron-methyl	504	182	29	10	83	81	10	101	10
169	Mesotrione	357	340.1	12	18	228.1	28	12	30	10
170	Metaflumizon	507.1	178	33	10	287	35	16	151	10
171	Methabenzthiazuron	222.1	165	23	10	150	43	8	46	10
172	Methfuroxam	230	137	27	8	111.1	21	8	81	10

No.	Compound	Precursor ion (m/z)	Quantification			Confirmation			DP(V)	EP(V)
			Product ion (m/z)	CE (V)	CXP (V)	Product ion (m/z)	CE (V)	CXP (V)		
173	Methiocarb	226.1	169	13	10	121	25	6	41	10
174	Methiocarb-sulfon	258	122	25	6	201.1	13	4	81	10
175	Methiocarb-sulfoxid	242	185	19	10	122.1	39	8	56	10
176	Methomyl	163	88	13	10	105.9	13	12	6	10
177	Methoprotryne	272.1	198.1	31	12	240.1	25	12	81	10
178	Methoxyfenozide	369.1	149	23	8	133	34	7	46	10
179	Metobromuron	259	169.9	25	10	148	21	8	66	10
180	Metolcarb	166	109.1	15	8	94	41	14	26	10
181	Metosulam	418	175	33	10	140	71	8	106	10
182	Metoxuron	229	72	21	8	46.1	37	8	76	10
183	Metsulfuron-methyl	382	167	21	10	198.9	29	12	61	10
184	Mexacarbate	223.1	166	19	8	151.1	31	8	21	10
185	Monocrotophos	224	127	23	6	98.1	17	8	41	10
186	Monolinuron	215	125.9	23	6	99	45	12	61	10
187	Monuron	199.1	71.9	21	10	126	33	6	76	10
188	Moxidectin	640.3	528.2	13	26	199.1	33	10	80	10
189	Naled	378.7	127	16	6.5	109	36	6.5	24	10
190	Neburon	275	88.1	21	10	114.1	21	8	101	10
191	Nicosulfuron	411.1	182	27	10	213	23	12	86	10
192	Nitenpyram	271.1	126	37	8	237	25	11	61	10
193	Norflurazon	304	283.9	33	16	160	43	10	131	10
194	Novaluron	493	158.1	25	10	141	65	8	96	10
195	Omethoate	214	182.9	15	10	124.9	29	6	46	10
196	Oxadiargyl	341.1	223	20	10	150.9	26	15	50	10
197	Oxadixyl	279.1	219.1	15	12	133.1	29	8	46	10
198	Oxamyl	237	72	31	8	90	11	8	25	10
199	Oxamyl oxime	163	72.1	17	6	90	23	14	46	10
200	Oxathiapiprolin	540.1	162.9	50	10	499.9	50	10	48	10
201	Oxycarboxin	268	174.9	21	10	147	31	10	66	10

No.	Compound	Precursor ion (m/z)	Quantification			Confirmation			DP(V)	EP(V)
			Product ion (m/z)	CE (V)	CXP (V)	Product ion (m/z)	CE (V)	CXP (V)		
202	Oxydemeton-methyl	247	169	19	10	124.9	29	6	41	10
203	Pencycuron	329	124.9	29	6	218	31	6	70	10
204	Penflufen	318.124	234.1	23	12	141	43	8	76	10
205	Penthiopyrad	360.049	276	21	14	176.9	43	10	71	10
206	Penoxsulam	484	195	5	30	164	5	30	10	10
207	Petoxamid	296.1	131.1	27	6	250	17	14	11	10
208	Phenmedipham	301.1	168.1	13	10	136	29	8	76	10
209	Phorate-sulfoxide	277	198.9	13	12	142.9	27	8	31	10
210	Phorate sulfone	293	171	15	8	96.8	47	14	51	10
211	Phosmet oxon	302.1	160.1	21	8	133.1	51	6	61	10
212	Phosphamidon	300.2	174	21	10	127.1	29	12	66	10
213	Phoxim	299	77	43	12	129	15	6	46	10
214	Picolinafen	377	238	37	12	359	27	18	106	10
215	Pinoxaden	401.3	317.1	53	4	57.1	29	18	91	10
216	Piperonyl butoxide	356.2	177	17	10	119.1	47	8	26	10
217	Pirimicarb-desmethyl	225.1	72	33	12	168	19	10	56	10
218	Prallethrin	301.3	105.1	31	6	123	23	8	66	10
219	Primisulfuron-methyl	469	254	29	14	199	31	10	76	10
220	Procyazine	253	226	23	15	186	20	11	40	10
221	Profoxydim	468.1	280.1	23	14	106.9	81	12	106	10
222	Promecarb	208.1	151	13	8	109.1	23	8	26	10
223	Prometon	226.1	142	31	8	184.1	25	10	81	10
224	Prometryn	242.09	158	31	10	200.1	25	12	76	10
225	Propamocarb	189.2	102.1	25	8	74	35	12	61	10
226	Propoxycarbazone-sodium	421.2	180.1	21	10	138.1	39	8	61	10
227	Proquinazid	373	288.9	33	16	330.9	19	18	71	10
228	Prosulfuron	420	141	25	8	167	25	10	76	10
229	Prothioconazole-desthio	312	69.9	61	8	125	37	6	96	10
230	Pymetrozine	218.1	104.9	27	12	78	59	12	81	10

No.	Compound	Precursor ion (m/z)	Quantification			Confirmation			DP(V)	EP(V)
			Product ion (m/z)	CE (V)	CXP (V)	Product ion (m/z)	CE (V)	CXP (V)		
231	Pyracarbolid	218.1	125	25	6	97	36	6	21	10
232	Pyraflufen-ethyl	413	338.9	5	17	289	5	30	30	10
233	Pyrethrins	317.2	149.0	15	8	107.1	27	6	61	10
234	Pyridafol	207	77	30	10	103.9	22	10	46	10
235	Pyridalil	489.9	108.9	29	6	183	26	10	101	10
236	Pyridate	379.1	206.9	23	12	350.9	15	20	51	10
237	Pyriofenone	365.8	184.0	30	12	209	33	14	40	10
238	Pyroxsulam	435	194.9	5	35	257.8	5	35	25	10
239	Quinclorac	242,0	223,9	21	14	161	53	10	41	10
240	Quinmerac	222.1	203.9	21	14	206	23	12	41	10
241	Quinoclamine	222.1	203.9	21	14	206	23	12	36	10
242	Rimsulfuron	432	182	29	10	325	21	18	76	10
243	Rotenone	395.1	213	31	12	192.1	33	10	136	10
244	Secbumeton	226.1	170.1	25	10	100	39	12	81	10
245	Sedaxane	332.1	159	5	31	139	5	33	56	10
246	Siduron	233.1	137	23	8	94.1	29	8	96	10
247	Silthiofam	268	139	26	14	73	45	14	122	10
248	Simetryn	214.1	124.1	27	8	144	28	7	86	10
249	Spinetoram J	748.9	142.1	29	10	97.9	60	10	44	10
250	Spinetoram L	760.8	142.1	28	10	98.2	65	10	44	10
251	Spinosyn A	732.4	142.1	35	8	98	103	16	136	10
252	Spinosyn D	746.4	142	35	8	98.1	101	16	146	10
253	Spirotetramat	374.1	302	23	16	330.1	21	20	131	10
254	Sulcotrione	329	139.1	18	10	69.2	38	10	36	10
255	Sulfentrazone	387	306.9	29	16	145.9	55	8	146	10
256	Sulfometuron-methyl	365	150	23	8	107	59	12	66	10
257	Sulfosulfuron	471	211	19	12	261	23	12	76	10
258	Tebufenozide	353.1	133.1	25	6	297.1	11	16	41	10
259	Tebuthiuron	229.08	172.1	25	10	116	35	8	81	10

No.	Compound	Precursor ion (m/z)	Quantification			Confirmation			DP(V)	EP(V)
			Product ion (m/z)	CE (V)	CXP (V)	Product ion (m/z)	CE (V)	CXP (V)		
260	Teflubenzuron	381	158	21	10	141	49	8	81	10
261	Tembotrione	458	340.9	23	20	262	53	14	56	10
262	Tepraloxydim	342.11	250.1	19	14	166	29	10	86	10
263	Terbumeton	226	170	25	10	114.1	33	8	81	10
264	Terbutryn	242.1	186.1	25	10	91	35	10	76	10
265	TFNA	192	79	51	4	98	43	4	76	10
266	TFNG	249	203	27	12	98	61	4	76	10
267	Thiabendazole	202	175	37	10	131.1	45	8	121	10
268	Thiacloprid	253	126	29	6	72.9	81	8	96	10
269	Thiamethoxam	292	211	17	12	181	31	10	61	10
270	Thidiazuron	221.1	102	21	12	127.9	23	12	61	10
271	Thiencarbazone-methyl	391.2	359	5	15	130	5	35	71	10
272	Thifensulfuron-methyl	388	167	21	8	204.9	35	12	61	10
273	Thiobencarb	258.1	125	25	6	89	69	10	36	10
274	Thiodicarb	355	88	27	10	108	21	8	51	10
275	Thiofanox-sulfone	251.1	57.2	15	10	75.9	15	10	50	10
276	Thiofanox-sulfoxide	252.1	104	16	9	57	35	8	50	10
277	Thiophanate-ethyl	371	150.9	27	8	325	17	17	71	10
278	Thiophanate-methyl	343	151	27	8	192	21	10	81	10
279	Topramezone	364	334	16	10	125	29	10	31	10
280	Tralkoxydim E	330.1	284.1	17	14	138	27	6	76	10
281	Tralkoxydim Z	330.1	284.1	17	14	138	27	6	76	10
282	Transfluthrin	388.2	163	41	8	163.2	37	8	36	10
283	Triasulfuron	402	167.1	23	10	141	27	8	81	10
284	Triazoxide	248	95	36	7	68	35	8	141	10
285	Tribenuron-methyl	396	155	19	8	181	27	10	76	10
286	Trichlorfon	257	108.9	27	18	220.8	17	14	61	10
287	Tricyclazole	190	163	31	10	136	39	8	96	10
288	Tridemorf	298	130	23	10	98	23	10	10	10

No.	Compound	Precursor ion (m/z)	Quantification			Confirmation			DP(V)	EP(V)
			Product ion (m/z)	CE (V)	CXP (V)	Product ion (m/z)	CE (V)	CXP (V)		
289	Triflumuron	359	156	21	10	139	45	8	56	10
290	Triflusulfuron-methyl	493	264	29	14	238	35	12	76	10
291	Triforine	434.91	389.8	17	20	98	43	14	60	10
292	Trinexapac-ethyl	253.1	207.1	19	12	68.9	33	12	80	10
293	Tritosulfuron	446	195	18	10	145	34	10	36	10
294	Uniconazole	292.1	70	59	10	125	37	8	106	10
295	Vamidothion	288	146	17	8	117.9	33	9	36	10
296	Valifenalate	399.2	155	5	47	161.1	5	33	76	10
297	Atrazine – d5 (IS)	221.1	179.1	25	10	69	101	10	51	10
298	Carbendazim – d3 (IS)	195	160	25	10	132	20	10	66	10
299	Izoproturon – d6 (IS)	213.1	78.3	27	14	171.2	21	10	66	10

CE-collision energy, CXP-cell exit potential , DP-declustering potential, EP-entrance potential

b) GC/MS/MS

No.	Compound	MRM transitions m/z (collision energy eV)		
		**Quantitative ion pairs**	**Qualitative ion pairs I**	**Qualitative ion pairs II**
1.	2,6-dichlorobenzamide	173 >145 (15)	189 > 173 (5)	173 > 109 (30)
2.	2-phenylphenol	169 > 141 (15)	169 > 115 (25)	170 > 141 (25)
3.	Acephate	136 > 94 (10)	125 > 47 (15)	142 > 95 (5)
4.	Acetochlor	174 > 146 (10)	146 > 131 (10)	223 > 132 (20)
5.	Acrinathrin	207 > 181 (10)	181 > 152 (30)	289 > 93 (10)
6.	Aldrin	263 > 193 (35)	255 > 220 (20)	263 > 191 (35)
7.	alpha-cypermethrin	163 > 127 (5)	163 > 91 (10)	165 > 91 (10)
8.	Amitraz	132 > 117 (15)	162 > 132 (5)	162 > 121 (10)
9.	Atrazine	215 > 58 (10)	215 > 200 (5)	200 > 122 (5)
10.	Atrazine-desethyl	186>172 (5)	172>94 (15)	172>69 (20)
11.	Azaconazole	217 > 173 (15)	219 > 175 (15)	173 > 145 (15)
12.	Azinphos - ethyl	132 > 77 (15)	160 > 77 (20)	160 > 132 (10)
13.	Azinphos - methyl	160 > 132 (10)	132 > 77 (15)	160 > 77 (20)
14.	Azoxystrobin	344 > 329 (15)	344 > 172 (40)	344 > 183 (25)
15.	Benalaxyl	266 > 148 (5)	148 > 105 (20)	148 > 77 (35)
16.	Benfluralin	292>264 (5)	292>206 (10)	263>206 (10)
17.	Beta cyfluthrin	163 > 91 (15)	163 > 127 (5)	199 > 170 (25)
18.	Bifenthrin	181 > 166 (10)	181 > 165 (25)	166 > 165 (20)
19.	Biphenyl	154>153 (15)	155>154 (15)	153.1>152 (15)
20.	Bitertanol	170 > 141 (20)	170 > 115 (40)	168 > 70 (10)
21.	Boscalid	140 > 76 (15)	140 > 112 (10)	112 > 76 (15)
22.	Bromophos - ethyl	359 > 303 (15)	303 > 285 (15)	242 > 97 (30)
23.	Bromophos - methyl	125 > 47 (10)	331 > 316 (15)	125 > 79 (5)
24.	Bromopropylate	183 > 155 (15)	185 > 157 (15)	339 > 183 (20)
25.	Bromuconazole	173 > 145 (15)	173 > 109 (30)	175 > 147 (15)
26.	Bupirimate	273 > 193 (5)	273 > 108 (15)	208 > 165 (10)
27.	Buprofezin	105 > 104 (10)	105 > 77 (20)	119 > 91 (15)
28.	Butralin	266>220 (10)	266>174 (20)	224>132 (15)
29.	Cadusafos	159 > 97 (15)	159 > 131 (5)	158 > 97 (15)
30.	Captafol	150 > 79 (5)	183 > 79 (10)	150 > 72 (5)
31.	Captan	151 > 80 (5)	151 > 79 (15)	149 > 79 (10)
32.	Carbaryl	144 > 115 (20)	144 > 116 (10)	116 > 115 (10)
33.	Carbofuran	164 > 149 (10)	149 > 121 (5)	149 > 77 (30)
34.	Carbosulfan	164 > 149 (10)	118 > 76 (5)	164 > 103 (25)
35.	Carboxin	235 > 143 (10)	235 >87 (20)	143 > 87 (5)
36.	Chinomethionate	233>206 (10)	233>148 (15)	206>148 (25)
37.	Chlorantraniliprole	239 > 214 (20)	277 > 243 (10)	277 >250 (10)
38.	Chlordan cis	271>236 (15)	372>265 (15)	374>265 (15)
39.	Chlordan trans	271>236 (15)	372>265 (15)	374>265 (15)
40.	Chlorfenapyr	136>102 (15)	246>227 (15)	327>246 (15)
41.	Chlorfenson	175>111 (10)	111>75 (15)	177>113 (10)
42.	Chlorfenvinphos	267 > 159 (15)	323 > 267 (10)	269 > 161 (15)
43.	Chlorobenzilate	139>111 (10)	251>139 (15)	139>75 (30)
44.	Chlorothalonil	264 > 168 (25)	264 > 229 (20)	266 > 231 (20)
45.	Chlorpropham	153 > 125 (10)	153 > 90 (25)	127 > 65 (25)
46.	Chlorpyrifos	314 > 258 (15)	199 > 171 (15)	197 > 169 (15)
47.	Chlorpyrifos - methyl	125 > 47 (15)	125 > 79 (5)	286 > 93 (20)
48.	Chlorthal-dimethyl	298>221 (25)	300>223 (25)	331>300 (10)
49.	Chlozolinate	186>154 (15)	188>147 (15)	186>109 (30)
50.	Clomazone	125 > 89 (15)	204 > 107 (20)	125 > 99 (15)
51.	Cyflufenamid	118 > 90 (10)	118 > 89 (25)	188 > 88 (35)
52.	Cyfluthrin	163 > 91 (15)	163 > 127 (5)	199 > 170 (25)
53.	Cypermethrin	163 > 127 (5)	163 > 91 (10)	165 > 91 (10)
54.	Cyphenothrin	123>81 (5)	181>152 (25)	208>141 (15)
55.	Cyproconazole	139 > 111 (15)	222 > 125 (15)	222 > 82 (10)
56.	Cyprodinil	225 > 224 (10)	224 > 208 (20)	226 > 225 (10)

No.	Compound	MRM transitions m/z (collision energy eV)		
		Quantitative ion pairs	Qualitative ion pairs I	Qualitative ion pairs II
57.	Deltamethrin	253 > 93 (15)	181 > 152 (25)	251 > 172 (5)
58.	Diazinon	199 > 93 (15)	137 > 84 (10)	137 > 54 (20)
59.	Dichlobenil	171 > 100 (25)	171 > 136 (15)	173 > 100 (25)
60.	Dichlofluanid	123 > 77 (20)	224 > 123 (10)	226 > 123 (10)
61.	Dichlorvos	109 > 79 (5)	185 > 93 (10)	145 > 109 (10)
62.	Diclofop-methyl	339>252 (10)	280>119 (10)	253>162 (15)
63.	Dicloran	206 > 176 (10)	160 > 124 (10)	124 > 73 (10)
64.	Dicofol	250 > 139 (20)	139 > 111 (20)	139 > 75 (20)
65.	Dicrotophos	127 > 109 (15)	127 > 95 (15)	193 > 127 (5)
66.	Dieldrin	277 > 241 (5)	263 > 193 (35)	263 > 191 (35)
67.	Diethyltoluamide (DEET)	119 > 91 (10)	119 > 65 (20)	91 > 65 (10)
68.	Difenoconazole	323 > 265 (15)	265 > 202 (20)	325 > 267 (15)
69.	Diflufenican (DFF)	266 > 238 (15)	266 > 246 (15)	394 > 266 (10)
70.	Dimethachlor	134 > 105 (10)	134 > 77 (25)	197 > 148 (10)
71.	Dimethenamid-P	230 > 154 (10)	154 > 111 (10)	232 > 154 (10)
72.	Dimethoate	86 > 46 (15)	93 > 63 (10)	87 > 86 (5)
73.	Dimethomorph	301 > 165 (10)	303 > 165 (10)	387 > 301 (10)
74.	Dimoxystrobin	205 > 116 (10)	116 > 89 (15)	116 > 63 (30)
75.	Dinikonazol	268 > 232 (10)	270 > 232 (10)	270 > 234 (10)
76.	Diphenylamine	169 > 168 (15)	168 > 167 (15)	167 > 166 (20)
77.	Dodemorph	169 > 168 (15)	168 > 167 (15)	167 > 166 (20)
78.	Endosulfan-sulphate	272 > 237 (15)	274 > 239 (15)	274 > 237 (15)
79.	Endrin	263 > 193 (35)	245 > 173 (30)	317 > 281 (5)
80.	EPN	169 > 141 (5)	169 > 77 (25)	185 > 157 (5)
81.	Epoxiconazole	192 > 138 (10)	192 > 111 (25)	165 > 138 (10)
82.	Esfenvalerate	167 > 125 (5)	209 > 141 (15)	181 > 152 (20)
83.	Etaconazole	173 > 145 (15)	173 > 109 (30)	245 > 55 (10)
84.	Ethion	153 > 97 (10)	125 > 97 (10)	231 > 175 (10)
85.	Ethofumesate	207 > 161 (5)	207 > 137 (10)	161 > 105 (10)
86.	Ethoprophos	158 > 97 (15)	158 > 114 (5)	139 > 97 (5)
87.	Etofenprox	163 > 135 (10)	163 > 107 (20)	135 > 107 (10)
88.	Etoxazole	141 > 113 (15)	141 > 63 (30)	204 > 176 (10)
89.	Famoxsadone	197 > 141 (15)	224 > 196 (10)	197 > 115 (30)
90.	Fenamidon	238 > 237 (10)	268 > 180 (20)	238 > 103 (15)
91.	Fenamiphos	154 > 139 (10)	217 > 202 (10)	303 > 288 (10)
92.	Fenarimol	219 > 107 (10)	251 > 139 (10)	139 > 75 (30)
93.	Fenazaquin	145 > 117 (10)	160 > 145 (5)	160 > 117 (20)
94.	Fenbuconazole	129 > 102 (15)	198 > 129 (5)	129 > 78 (20)
95.	Fenchlorphos	285 > 270 (15)	288 > 272 (15)	125 > 47 (15)
96.	Fenitrothion	125 > 47 (15)	125 > 79 (5)	277 > 260 (5)
97.	Fenoxaprop-P-ethyl	361 > 288 (10)	288 > 91 (20)	288 > 119 (10)
98.	Fenoxycarb	255 > 186 (10)	186 > 158 (5)	185 > 129 (5)
99.	Fenpropathrin	181 > 152 (25)	208 > 181 (5)	125 > 55 (10)
100.	Fenpropidin	98 > 55 (15)	98 > 70 (10)	117 > 91 (15)
101.	Fenpropimorph	128 > 70 (10)	128 > 110 (5)	128 > 86 (10)
102.	Fenpyrazamine	331>230 (5)	230>132 (10)	-
103.	Fenthion	278 > 109 (15)	125 > 47 (10)	124.9 > 79 (5)
104.	Fenvalerate	167>125 (5)	208>141 (15)	181>152 (20)
105.	Fipronil	351 > 255 (15)	367 > 213 (25)	255 > 228 (15)
106.	Flonicamid	174 > 146 (10)	174 > 126 (30)	146 > 69 (30)
107.	Fluazifop-p-butyl	282 > 91 (20)	282 > 238 (20)	383 > 282 (10)
108.	Fludioxonil	248 > 127 (30)	248 > 182 (10)	248 > 154 (20)
109.	Flufenacet	151 > 136 (10)	151 > 95 (30)	123 > 95 (20)
110.	Flumetralin	175>111 (10)	111>75 (15)	177>113 (10)
111.	Fluopicolide	209 > 182 (10)	209 > 146 (20)	347 > 172 (20)
112.	Fluquinconazole	340 > 298 (15)	108 > 57 (15)	340 > 108 (40)
113.	Flurochloridone	187 > 159 (10)	311 > 174 (15)	145 > 95 (15)

No.	Compound	MRM transitions m/z (collision energy eV)		
		Quantitative ion pairs	Qualitative ion pairs I	Qualitative ion pairs II
114.	Fluroxypyr-1-methylheptyl	209 > 181 (10)	237 >181 (15)	237 > 209 (5)
115.	Flurtamone	333 > 120 (15)	199 > 157 (20)	157 > 137 (15)
116.	Flusilazole	233 > 165 (15)	233 > 91 (20)	315 > 233 (10)
117.	Flutolanil	173 > 145 (15)	281 > 173 (10)	173 > 95 (30)
118.	Flutriafol	123 > 95 (15)	123 > 75 (25)	219 > 123 (15)
119.	Folpet	260 > 130 (15)	262 > 130 (15)	260 > 232 (5)
120.	Fonofos	136>109 (5)	108>80 (5)	108>62 (15)
121.	Formothion	170 > 93 (5)	125 > 47 (15)	125 > 79 (5)
122.	Fosthiazate	195 > 103 (5)	195 > 60 (20)	199 > 102 (5)
123.	Fuberidazole	184 > 156 (10)	184 > 155 (30)	183 > 155 (10)
124.	Haloxyfop-methyl	316 > 91 (20)	316 > 272 (20)	375 > 316 (10)
125.	Heptachlor	100 > 58 (10)	100 > 72 (5)	198 > 126 (5)
126.	Heptachlor endo- epoxide	183 > 155 (15)	183 > 119 (25)	217 > 182 (20)
127.	Heptenophos	124 > 89 (10)	124 > 63 (35)	109 > 79 (5)
128.	Hexachlorobenzene (HCB)	284 > 214 (30)	284 > 249 (15)	289 > 212 (30)
129.	Hexaconazole	256 > 82 (10)	231 > 175 (10)	256 > 159 (15)
130.	Hexythiazox	227 > 149 (10)	184 > 149 (10)	184 > 115 (20)
131.	Imazalil	215 > 173 (5)	217 > 175 (5)	173 > 145 (15)
132.	Imibenconazole	125 > 89 (20)	125 > 99 (20)	253 > 82 (5)
133.	Indoxacarb	203 > 134 (15)	203 > 106 (25)	203 > 78 (30)
134.	Ipconazole	167 > 152 (5)	167 > 125 (5)	249 > 125 (15)
135.	Iprodione	244 > 187 (5)	187 > 124 (25)	314 > 56 (20)
136.	Iprovalicarb	119 > 91 (15)	116 > 98 (5)	116 > 55 (15)
137.	Isocarbophos	120 > 92 (10)	136 > 108 (15)	121 > 65 (15)
138.	Isofenphos	213 > 121 (10)	213 > 185 (5)	185 > 121 (5)
139.	Isofenphos - methyl	199 > 121 (10)	199 > 167 (10)	167 > 123 (5)
140.	Isoprocarb	121 > 77(20)	136 > 121 (10)	121 > 103 (10)
141.	Isoprothiolane	162 > 85 (20)	162 > 134 (5)	231 > 189 (10)
142.	Krezoxim-methyl	116 > 89 (15)	116 > 63 (30)	131 > 89 (30)
143.	Lenacil	153 > 136 (15)	153 > 82 (20)	153 > 110 (15)
144.	Malaoxon	127 > 99 (5)	127 > 55 (5)	99 > 71 (5)
145.	Malathion	127 > 99 (5)	173 > 99 (15)	158 > 125 (5)
146.	Mecarbam	159 > 131 (5)	131 > 74 (5)	131 > 86 (10)
147.	Metalaxyl	234 > 146 (20)	220 > 192 (5)	234 > 174 (10)
148.	Metamitron	104 > 77 (15)	202 > 174 (5)	104 > 51 (35)
149.	Metazachlor	133 > 132 (10)	132 > 117 (15)	209 > 132 (15)
150.	Metconazole	125 > 89 (20)	125 > 99 (20)	138 > 69 (10)
151.	Methacrifos	208 > 180 (5)	125 > 47 (10)	125 > 79 (5)
152.	Methamidophos	141 > 95 (5)	95 > 79 (10)	95 > 64 (10)
153.	Methidathion	145 > 85 (5)	145 > 58 (15)	85 > 58 (5)
154.	Methoxychlor (DMDT)	227 > 169 (25)	227 > 141 (40)	227 > 212 (15)
155.	Metolachlor	238 > 162 (10)	162 > 133 (15)	240 > 162 (10)
156.	Metrafenone	209 > 166 (10)	395 > 365 (15)	227 > 169 (10)
157.	Metribuzin	198 > 82 (15)	198 > 55 (30)	144 > 128 (10
158.	Mevinphos	127 > 109 (10)	127 > 95 (15)	192 > 127 (10)
159.	Mirex	273>238 (15)	273>236 (15)	271>236 (15)
160.	Molinate	126>55 (10)	126>83 (5)	126>98 (5)
161.	Myclobutanil	179 > 125 (10)	179 > 90(30)	150 > 123 (15)
162.	Napropamide	128 > 72 (5)	128 > 100 (10)	271 > 72 (15)
163.	Nitrofen	202 > 139 (20)	283 > 253 (10)	283 > 202 (10)
164.	o.p' DDT	235 > 165 (20)	237 > 165 (20)	235 > 199 (15)
165.	Oxyfluorfen	252 > 196 (20)	252 > 146 (30)	300 > 223 (15)
166.	p,p' DDT	235 > 165 (20)	237 > 165 (20)	235 > 199 (15)
167.	p.p' DDD	235 > 165 (20)	237 > 165 (20)	235 > 199 (15)
168.	p.p' DDE	246 > 176 (30)	316 > 246 (15)	318 > 246 (15)
169.	Paclobutrazol	236 > 125 (10)	125 > 89 (20)	236 > 167 (10)
170.	Paraoxon	109 > 81 (10)	109 > 91 (5)	149 > 119 (5)

No.	Compound	MRM transitions m/z (collision energy eV)		
		Quantitative ion pairs	Qualitative ion pairs I	Qualitative ion pairs II
171.	Paraoxon methyl	109 > 79 (5)	230 > 106 (15)	230 > 136 (5)
172.	Parathion	139 > 109 (5)	291 > 109 (10)	139 > 81 (15)
173.	Parathion - methyl	269 > 109 (10)	125 > 47 (10)	125 > 79 (5)
174.	Pebulate	12>72 (5)	128>57 (10)	161>128 (5)
175.	Penconazole	248 > 192 (15)	248 > 157 (25)	159 > 89 (35)
176.	Pendimethalin	252>162 (25)	252 > 161 (15)	162 > 161 (10)
177.	Pentachloroaniline	262>192 (20)	264>194 (20)	266>194 (20)
178.	Permethrin	183 > 168 (10)	183 > 165 (10)	183 > 153 (15)
179.	Phenothrin	183>168 (10)	183>155 (5)	122>81 (5)
180.	Phenthoate	274 > 121 (10)	274 > 125 (15)	121 > 77 (25)
181.	Phorate	121 > 65 (10)	121 > 47 (30)	129 > 65 (15)
182.	Phosalone	182 > 111 (15)	182 > 102 (15)	182 > 75 (30)
183.	Phosmet	160 > 77 (20)	160 > 133 (10)	160 > 105 (15)
184.	Phthalimide	147>103 (5)	147>76 (25)	104>76 (10)
185.	Picoxystrobin	145 > 102 (25)	145 > 115 (15)	145 > 117 (10)
186.	Pirimicarb	238 > 166 (10)	166 > 55 (20)	166 > 96 (15)
187.	Pirimiphos - methyl	290 > 125 (20)	233 > 151 (5)	233 > 125 (5)
188.	Pirimiphos ethyl	318 > 166 (10)	318 > 182 (10)	152 > 84 (10)
189.	Prochloraz	196 > 97 (30)	180 > 138 (10)	310 > 70 (15)
190.	Procymidone	96 > 67 (10)	96 > 53 (15)	283 > 96 (10)
191.	Profenofos	208 > 63 (30)	339 >269 (15)	299 > 269 (5)
192.	Propachlor	120 > 77 (10)	176 > 57 (10)	120 > 92 (10)
193.	Propaquizafop	163 > 100 (25)	163 > 136 (10)	299 > 91 (20)
194.	Propargite	135 > 107 (10)	150 > 135 (5)	135 > 77 (30)
195.	Propazine	214 > 172 (10)	229 > 58 (10)	229 > 214 (5)
196.	Propham	119 > 91 (10)	137 > 93 (10)	179 > 93 (15)
197.	Propiconazole	173 > 145 (15)	173 > 109 (30)	173 > 74 (45)
198.	Propoxur	110 > 63 (25)	110 > 64 (15)	152 > 110 (10)
199.	Propyzamide	173 > 145 (15)	175 > 147 (15)	173 > 109 (30)
200.	Prosulfocarb	128 > 86 (0)	251 > 86 (10)	251 > 128 (10)
201.	Prothiofos	113 > 95 (10)	267 > 239 (5)	309 > 239 (15)
202.	Pyraclostrobin	132 >77.1 (20)	164 > 132 (10)	132 > 104 (15)
203.	Pyrazophos	221 > 193 (10)	232 > 204 (10)	221 > 149 (15)
204.	Pyridaben	147 > 117 (20)	147 > 132 (10)	147 > 105 (10)
205.	Pyrimethanil	198 > 183 (15)	198 > 118 (35)	198 > 158 (20)
206.	Pyriproxyfen	136 > 78 (20)	136 > 96 (15)	321 > 222 (10)
207.	Quinalphos	146 > 118 (10)	146 > 91 (30)	157 > 129 (15)
208.	Quinoxyfen	237 > 208 (30)	272 > 237 (10)	307 > 237 (20)
209.	Quintozene	295>265 (10)	295>237 (15)	265>237 (10)
210.	Quizalofop-P-ethyl	372 > 299 (10)	163 > 136 (10)	163 > 100 (20)
211.	Resmethrin	123>81 (5)	171>128 (5)	171>143 (15)
212.	Simazine	201 > 173 (5)	173 > 172 (5)	173 > 138 (5)
213.	Spirodiclofen	109 > 81 (10)	109 > 79 (15)	312 > 259 (10)
214.	Spiromesifen	272 > 524 (5)	272 > 209 (10)	273 > 255 (5)
215.	Spiroxamine	100 > 58 (10)	100 > 72 (5)	198 > 126 (5)
216.	Sulfotep	201>145 (10)	321>201 (10)	321>145 (25)
217.	Tebuconazole	125 > 89 (15)	250 > 125 (20)	125 > 99 (20)
218.	Tebufenpyrad	276 > 171 (10)	333 > 171 (15)	318 > 131 (15)
219.	Tecnazene	261 > 203 (20)	215 > 179 (5)	259 > 201 (5)
220.	Tefluthrin	177 > 127 (15)	197 > 141 (10)	177 > 87 (30)
221.	Terbacil	160>117.1 (5)	161>144 (10)	160>76 (15)
222.	Terbufos	230>175 (10)	230>129 (20)	152>97 (5)
223.	Terbufos sulfone	152> 96(10)	124>96 (5)	198>143 (10)
224.	Terbuthylazine	173 > 172 (5)	229 > 173 (5)	173 > 138 (5)
225.	Tetrachlorvinfos	329 > 109 (15)	331 > 109 (15)	109 > 79 (5)
226.	Tetraconazole	171 > 136 (10)	336 > 218 (20)	336 > 204 (30)

No.	Compound	MRM transitions m/z (collision energy eV)		
		Quantitative ion pairs	Qualitative ion pairs I	Qualitative ion pairs II
227.	Tetradifon	159 > 131 (10)	227 > 199 (15)	159 > 111 (20)
228.	Tetrahydrophthalimide (THPI)	79.0>77.0 (15)	79.0>51.0 (25)	151.0>80.0 (5)
229.	Tetramethrin	164 >107 (10)	164 > 77 (25)	123 > 81 (10)
230.	Thionazin	143>79 (10)	175>79 (10)	107>79 (15)
231.	Tolclofos - methyl	265 > 250 (15)	265 > 93 (25)	125 > 47 (15)
232.	Tolfenpyrad	211>210 (10)	197>154 (10)	383>171 (25)
233.	Tolylfluanid	137 > 91 (20)	238 > 137 (15)	137 > 65 (30)
234.	Tralomethrin	252>93 (15)	181>152 (25)	250>172 (5)
235.	Triadimefon	208 > 181 (5)	208 > 111 (20)	128 > 65 (20)
236.	Triadimenol	128 > 65 (25)	168 > 70 (10)	128 > 100 (10)
237.	Triallate	268>184 (20)	270>186 (20)	268>226 (10)
238.	Triazophos	161 > 134 (5)	161 > 106 (10)	161 > 91 (15)
239.	Trifloxystrobin	116 > 89 (15)	116 > 63 (30)	131 > 89 (15)
240.	Triflumizole	206 > 179 (15)	206 > 186 (10)	179 > 144 (15)
241.	Trifluralin	306 > 264 (5)	264 > 160 (15)	264 > 206 (5)
242.	Triticonazole	235 > 217 (5)	235 > 182 (10)	217 > 165 (25)
243.	Vinclozolin	125 > 47 (15)	125 > 79 (5)	286 > 93 (20)
244.	zeta-cypermethrin	163 > 127 (5)	163 > 91 (10)	165 > 91 (10)
245.	Zoxamide	187 > 159 (15)	189 > 161 (15)	187 > 123 (30)
246.	α-endosulfan	195 > 159 (5)	195 > 160 (5)	195 > 125 (20)
247.	α-HCH	217 > 181 (5)	219 > 183 (5)	181 > 145 (15)
248.	β-endosulfan	207 > 172 (15)	195 > 159 (10)	195 > 125 (25)
249.	β-HCH	181 > 145 (15)	217 > 181 (5)	219 > 183 (5)
250.	γ- cyhalothrin	197 > 141 (10)	181 > 152 (25)	197 > 161 (5)
251.	γ-HCH (lindane)	217 > 181 (5)	181 > 145 (15)	219 > 183(5)
252.	δ-HCH	217>181 (5)	181>145 (15)	219 >183 (5)
253.	λ-cyhalothrin	197 > 141 (10)	181 > 152 (25)	197 > 161 (5)
254.	τ-fluvalinate	250 > 55 (40)	181 > 152 (40)	250 > 200 (40)
255.	Triphenyl phosphate (TPP) (IS)	215 > 168 (15)	232 > 215 (10)	326 > 325 (5)

Table S4. Results of participation in proficiency testing in fruit and vegetables.

	Pesticide	Assigned value [mg kg^{-1}]	Labolatory results [mg kg^{-1}]	z-score
Banana Homogenate<br>EUPT-FV26, European Commission - University of Almeria (Spain), 2024				
1	Ametoctradin	0.078	0.084	0.3
2	Azoxystrobin	0.461	0.45	-0.1
3	Bifenthrin	0.166	0.17	0.1
4	Chlorpyrifos	0.052	0.058	0.4
5	Cypermethrin	0.157	0.17	0.3
6	Diazinon	0.079	0.088	0.4
7	Flupyradifurone	0.0166	0.17	0.1
8	Fluquinconazole	0.064	0.082	1.1
9	Fluxapyroxad	0.478	0.45	-0.2
10	Monocrotophos	0.059	0.053	-0.4
11	Myclobutanil	0.087	0.078	-0.4
12	Omethoate	0.096	0.12	1.0
13	Pyrimethanil	0.098	0.089	-0.4
14	Spiroxamine	0.224	0.22	-0.1
15	Thiabendazole	0.890	0.89	0.0
16	Metconazole	0.090	0.076	-0.6

	Pesticide	Assigned value [mg kg^{-1}]	Labolatory results [mg kg^{-1}]	z-score
Melon Homogenate<br>EUPT-FV25, European Commission - University of Almeria (Spain), 2023				
1	Chlorpyrifos-methyl	0.090	0.069	-0.9
2	Cyazofamid	0.083	0.081	-0.1
3	Diazinon	0.104	0.077	-1.0
4	Difenoconazole	0.222	0.24	0.3
5	Ethirimol	0.388	0.25	-1.4
6	Fenazaquin	0.078	0.072	-0.3
7	Fenitrothion	0.085	0.062	-1.1
8	Flutriafol	0.304	0.29	-0.2
9	Imazalil	0.086	0.067	-0.9
10	Mandipropamid	0.451	0.48	0.3
11	Mepanipyrim	0.081	0.084	0.1
12	Metrafenone	0.562	0.42	-1.0
13	Profenofos	0.117	0.11	-0.2
14	Proquinazid	0.079	0.058	-1.1
15	Pyriproxyfen	0.245	0.18	-1.1
16	Thiamethoxam	0.208	0.17	-0.7
17	Zoxamide	0.457	0.42	-0.3
18	Novaluron	0.102	0.085	-0.7

	Pesticide	Assigned value [mg kg^{-1}]	Labolatory results [mg kg^{-1}]	z-score
Tomato homogenate<br>EUPT-SRM 17, European Commission– National Food Institute Technical University of Denmark, 2022				
1	Captan	0.172	0.20	0.7
2	Chlorotalonil	0.151	0.19	1.0
3	Dithiocarbamates	0.187	0.17	-0.4
4	Dodine	0.100	0.12	0.8
5	Emamectin B1a	0.046	0.055	0.8
6	Folpet	0.249	0.29	0.7
7	Pymetrozine	0.150	0.095	-1.5

	Pesticide	Assigned value [mg kg^{-1}]	Labolatory results [mg kg^{-1}]	z-score
Aubergine homogate<br>EUPT-FV23, European Commission - University of Almeria (Spain), 2021				
1	Acetamipryd	0.175	0.174	0.0
2	Chlorfenapyr	0.299	0.26	-0.5
3	Chlorpiryfos	0.070	0.057	-0.7
4	Clofentezine	0.096	0.079	-0.7
5	Diazinon	0.755	0.58	-0.9
6	Dimethoate	0.079	0.072	-0.4
7	Endosulfan sulfate	0.283	0.16	-1.7
8	Fenarimol	0.319	0.259	-0.8
9	Flonicamid	0.102	0.097	-0.2
10	Imazalil	0.187	0.13	-1.2

11	Methomyl	0.226	0.23	0.1
12	Quinoxyfen	0.193	0.14	-1.1
13	Spinosad	0.196	0.159	-0.8
14	Tau-Fluvalinate	0.130	0.152	0.7
15	Tetraconazole	0.156	0.132	-0.6
16	Thiabendazole	0.196	0.16	-0.7
17	Triazophos	0.210	0.162	-0.9
18	Zoxamide	0.156	0.12	-0.9

	Pesticide	Assigned value [mg kg^{-1}]	Labolatory results [mg kg^{-1}]	z-score
Onion homogenate<br>EUPT-FV22, European Commission - University of Almeria (Spain), 2020				
1	Azoxystrobin	1.156	1.51	1.2
2	Chlorpropham	0.229	0.321	1.6
3	Cyprodinil	0.289	0.361	1.0
4	Diazinon	0.079	0.084	0.3
5	Dicloran	0.104	0.115	0.4
6	Dimethomorph	0.275	0.301	0.4
7	Fenamidone	0.185	0.197	0.3
8	Fenhexamid	0.568	0.39	-1.3
9	Fludioxonil	0.199	0.171	-0.6
10	Fluopicolide	0.605	0.51	-0.6
11	Fluopyram	0.044	0.039	-0.5
12	Fluxapyroxad	0.069	0.091	1.3
13	Tebuconazole	0.051	0.05	-0.1
14	Tefluthrin	0.047	0.056	0.8
15	Triadimenol	0.032	0.035	0.3
